# Effects of the Dental Caries Preventive Procedure on the White Spot Lesions during Orthodontic Treatment—An Open Label Randomized Controlled Trial

**DOI:** 10.3390/jcm11030854

**Published:** 2022-02-06

**Authors:** Yudai Shimpo, Yoshiaki Nomura, Toshiko Sekiya, Chihiro Arai, Ayako Okada, Kaoru Sogabe, Nobuhiro Hanada, Hiroshi Tomonari

**Affiliations:** 1 Department of Orthodontics, Tsurumi University School of Dental Medicine, Yokohama 230-8501, Japan; PD20006@stu.tsurumi-u.ac.jp (Y.S.); sekiya-t@tsurumi-u.ac.jp (T.S.); arai-chihiro@tsurumi-u.ac.jp (C.A.); tomonari-h@tsurumi-u.ac.jp (H.T.); 2 Department of Translational Research, Tsurumi University School of Dental Medicine, Yokohama 230-8501, Japan; sogabe-k@tsurumi-u.ac.jp; 3 International Photocatalyst Research Institute, University of Shanghai for Science and Technology, 516 Jungong Road, Shanghai 200093, China; gtfgene@gmail.com; 4 Department of Operative Dentistry, Tsurumi University School of Dental Medicine, Yokohama 230-8501, Japan; okada-a@tsurumi-u.ac.jp

**Keywords:** tooth surface disinfection, quantitative light-induced fluorescence, mixed effect model, fixed orthodontic treatment, oral microbiome

## Abstract

(1) Background: The aim of this study was to assess the preventive effect of tooth surface disinfection treatment, in addition to fluoride application, during fixed orthodontic treatment. (2) Methods: An open label randomized control trial for the evaluation of the dental caries preventive procedure was performed for the patients with high caries risk who had been visited at Department of Orthodontics, Tsurumi University Dental Hospital for orthodontics treatment. The follow-up period was six months. White spot lesions (WSLs) were evaluated by quantitative light-induced fluorescence (QLF). Cariogenic bacteria were monitored and evaluated by bacterial culture. In addition, the oral microbiome was evaluated by a next-generation sequence (NGS). (3) Results: By the mixed effect modeling, tooth surface disinfection treatment significantly reduced cariogenic bacteria and all parameters obtained by QLF. (4) Conclusions: Tooth surface disinfection treatment, in addition to PMTC and fluoride application, were effective for dental caries prevention and keeping a healthy microbiome during orthodontic treatment.

## 1. Introduction

One of the goals of orthodontic treatment is to correct malocclusion and deformities to improve dental esthetics [1]. Despite the post-therapeutic esthetic advantages of orthodontics, the treatment is associated with an increase in dental caries incidence since orthodontic brackets tend to retain dental biofilm [2] and interfere with proper tooth cleaning [3,4].

Fixed orthodontic appliances are a common and effective tool to treat malocclusion. However, they have side effects such as a change of the composition of oral microbiome [5,6]. The complicated undercut shape of fixed orthodontic appliances makes teeth cleaning more difficult and can induce dental plaque accumulation [2,4]. Subsequently, restorative treatment is necessary in some cases. It has been suggested that the risk of white spot lesions (WSLs), dental caries, and periodontal complication are caused by the change in oral microbiome [7]. The incidence of WSLs in patients undergoing fixed orthodontic treatment is high [8]. However, incipient carious lesions can be remineralized by fluoride application [9,10]. Previous studies have suggested that an increase in *mutans streptococci*, regarded as a major risk factor for dental caries, is associated with placement of fixed orthodontic appliances [11,12].

Various prevention methods for demineralization have been proposed such as fluoride application [13,14] and the use of antimicrobial agents. For example, chlorhexidine (CHX) [15,16] and application of casein phosphopeptide containing agents were effective for the prevention of WSLs [17]. Many studies evaluated the effect of WSLs treatment at a subject level and tooth level [18,19,20]. Only a few studies evaluated the effect at site level [21]. Although various factors affect caries risk, high levels of mutans streptococci and poor oral hygiene are major risk factors. The use of antimicrobial agents reduce cariogenic activity by altering composition of pathogenic bacteria in dental biofilm. This ecological modification of the biofilm may balance the process of demineralization and remineralization between the dental surface and the adjacent plaque [15,19,22]. Among the antimicrobial agents available for dental use, CHX, which is particularly sensitive to mutans streptococci, is the most commonly used to control cariogenic activity.

Conventional methods of bacteriological identification such as cultivation present limitations to the analysis of microbial community structure and diversity since the human bacterial flora contains many unculturable species. Next generation sequencing (NGS) technology has enabled analysis and comparison of bacterial composition, including unculturable bacteria. However, there are few reports concerning the relationship between orthodontic treatment and the dynamics of the oral microbiome [23,24,25,26,27]. In addition, these reports only analyzed periodontal pathogens [25,26,27].

The aim of this study was to assess the preventive effect of antimicrobial treatment in addition to fluoride application during fixed orthodontic treatment. Outcome variables were set as evaluating the changes the WSLs at site level by using the quantitative light-induced fluorescence system (QLF). In addition, oral microbiome dynamics were also evaluated.

## 2. Materials and Methods

### 2.1. Trial Design

An open label randomized control trial for the evaluation of the dental caries preventive procedure was performed for the patients with high caries risk who had been visited at Department of Orthodontics, Tsurumi University Dental Hospital for orthodontics treatment. The follow-up period was six months. WSLs were evaluated by the Quantitative Light-induced Fluorescence. Cariogenic bacteria were monitored and evaluated by bacterial culture. In addition, the oral microbiome was evaluated by a next-generation sequence (NGS).

### 2.2. Setting

From October 2020 to October 2021, thirty-two patients under orthodontic treatment by fixed orthodontic appliance were recruited. For these patients, oral examination, and caries risk assessment by the caries management by risk assessment (CAMBRA) was carried out as screening [28,29]. Four patients were excluded due to a medium or low caries risk by CAMBRA. For the rest of the 28 patients, salivary levels of cariogenic bacteria were evaluated. Among them, six were excluded due to the low proportion of mutans streptococci (less than 0.1%) or transferred to another hospital due to changes in employment. A total of 22 (8 males, 14 females) patients were finally included.

### 2.3. Inclusion Criteria

The inclusion criteria were as follows: (1) age range, 13–35 years; (2) patients undergoing fixed orthodontic treatment after leveling and alignment stage of front teeth; (3) caries risk assessment by CAMBRA indicates high or extreme; (4) salivary levels of mutans streptococci over 0.2% against total streptococci; (5) not using antibiotics for at least six weeks before saliva sampling or during the experimental period [19,22,30,31,32].

Patients who were ASA Class II or below according to the ASA Physical Status Classification System [33] were excluded.

### 2.4. Sample Size Calculation

According to the mean values and standard deviations of the previous studies [34], the sample size to obtain the tooth level intragroup significant difference was from 22 to 105 teeth (α = 0.05 and power = 0.80). In addition, in vitro study had shown that 84 teeth in each group were required to obtain a significant difference in intragroup comparisons [20]. In addition, by the results of this study, to obtain the significant difference of ΔQ value by the application of CHX or fluoride, either six or four teeth was necessary (α = 0.05 and power = 0.80).

This study was conducted on a total of 231 teeth by 22 subjects (110 teeth for Tooth surface disinfection group and 121 teeth for fluoride application group). The sample size was considered to be enough.

### 2.5. Randomization

This study was a single-center, open-label, two-arm parallel, randomized clinical trial with a 1:1 allocation ratio. Random sequence generation was performed with a computerized random 1:1 allocation using block sizes of 2. Randomization was performed on a computer with SPSS Statistics Ver 27.0 (IBM, Tokyo, Japan) by Y.S. No changes were made to the protocol after the trial commencement. After screening, the groups were created and locked before the start of the study. Patients were randomly allocated to the fluoride application group as a control (Group 1) or the tooth surface disinfection group as a test (Group 2).

To ensure confidentiality in allocation, linkable anonymizing was carried out and correspondence table was stored in the computer which was not connected to the network. This computer was put in the locked room at Department of Orthodontics, Tsurumi University Dental Hospital, Kanagawa, Japan. Figure S1 shows a flowchart of the patients’ allocation and dropout.

### 2.6. Clinical Procedure

Dental biofilm removal was performed by professional mechanical tooth cleaning (PMTC) for all patients. After PMTC, patients in Group 1 participated in a program to prevent dental caries as is normally conducted in the Department of Orthodontics, Tsurumi University Dental Hospital (tooth brushing instruction and topical application of 9000 ppm fluoride (Fluor-Gel, BEE BRAND MEDICO DENTAL.CO., LTD., Osaka, Japan)). All patients were given oral hygiene instruction at the first visit and instructed to use a fluoride-containing dentifrice twice a day.

For patients in Group 2, custom made individual tray was fabricated for tooth surface disinfection (Dental Drug Delivery System; 3DS) treatment. After PMTC, an impression was taken and individual tray was fabricated by using Erkopress (Erkodent, Pfalzgrafenweiler, Germany) on the gypsum cast. Afterwards, the Plak Out (KerrHawe SA, Bioggio, Switzerland), a commercially available 0.2% CHX gel with specifically antibacterial action against mutans streptococci, was poured into the individual tray and held against the surface of teeth for 5 min. After this procedure, the patients washed their mouth with water and applied 9000 ppm fluoride (Fluor-Gel, BEE BRAND MEDICO DENTAL.CO. LTD., Osaka, Japan) to the tooth surface. Tooth surface disinfection treatment applied at the start of the study (T0) and after three months (T3).

### 2.7. Outcome Variables

#### 2.7.1. Assessment of WSLs

The primary endpoint of this study was to evaluate the quantitative changes of enamel demineralization using the QLF method. The primary outcome was that the changes in the level of fluorescence (ΔF (%) and ΔF_max_ (%)), the area of the lesion (WS area(mm^2^)) and the percentage loss of fluorescence (ΔQ (% × mm^2^)). Tooth surfaces evaluated in this study were the labial surfaces of the central incisor, lateral incisor, and canine teeth. QLF images were obtained to assess WSLs in all patients at baseline (T0) and after 6 months (T6). The QLF images were analyzed by placing an analysis patch on the stained area dividing into nine sites, ensuring that the boundaries of the patch corresponded to sound enamel (Figure S2).

#### 2.7.2. Assessment of the Cariogenic Bacteria

The effect of tooth surface disinfection was monitored by salivary levels of cariogenic bacteria (mutans streptococci and *Lactobacillus*) through this study. Saliva samples were collected at screening (TS) and monthly during the study periods until six months later (T0, T1, T2, T3, T4, T5, and T6) by the test kit (BML dental laboratory, Saitama, Japan). The log_10_ transformed number of mutans streptococci and *Lactobacillus*, and the proportion of mutans streptococci against total streptococci were calculated by bacterial culture method. Additionally, saliva samples for microbiome analysis with a next-generation sequence (NGS) were analyzed at baseline (T0) and after 6 months (T6).

### 2.8. Next-Generation Sequence

#### 2.8.1. Microbial DNA Extraction

Saliva samples suspended in PBS were collected by centrifuging the sample at 3000 rpm for 10 min. DNA extraction was performed by the Maxwell 16 LEV Blood DNA Kit (Promega KK, Tokyo, Japan), according to the manufacturer’s instructions. DNA concentrations were measured by Nano Drop ND-2000 (Thermo Fisher Scientific KK, Tokyo, Japan). The degradation of DNA was visually checked by electrophoresis on a 1% agarose gel, and the contamination of RNA was checked using the Qubit dsDNA BR Assay Kit (Thermo Fisher Scientific KK, Tokyo, Japan). Samples that filled the following criteria were used for further sequence analysis: Concentration > 20 ng/μL; volume 20 μL; A260/280 1.8; and A260/230 > 1.5. In this study, all samples passed these criteria.

#### 2.8.2. Microbial Community Analysis

Extracted DNA was analyzed in the laboratory (Chun Lab, Seoul, Korea). Polymerase chain reaction (PCR) amplification was performed using primers specific to the V3–V4 region next-generation sequencing tags of the 16S rRNA gene in the extracted bacterial DNA. The taxonomic classification of each read was assigned based on a search of the EzBioCloud 16S database [35,36]. This database contains the 16S rRNA genes of strains that have valid published names and representative species-level phylotypes of both cultured and uncultured entries in the GenBank database, with complete hierarchical taxonomic classification from the phylum to the species levels.

#### 2.8.3. Bioinformatics Analysis

The number of 16S rRNA gene copies (absolute abundance) of operational taxonomic units (OTUs) was calculated by multiplying their respective relative abundance by the total number of 16S rRNA gene copies.

### 2.9. Statistical Analysis

#### 2.9.1. QLF Data Analysis

Descriptive statistics were calculated by SPSS Statistics ver 27.0 (IBM, Tokyo, Japan) and visualized by free software R ver 4.12.

#### 2.9.2. Mixed Effect Modeling

For the changes of parameters of WSLs at site level, mixed effect modeling was performed. Independent variables used for the modeling were Groups and time for patients’ level and tooth type for tooth level. Random intercepts were included in patients, tooth and site levels. Mixed effect modeling was also applied for the changes of dental caries and periodontal disease pathogens obtained by next generation sequence [37,38]. Models for QLF parameters and for oral pathogens are specified in File S1. Analysis was performed by SPSS Statistics ver27.0 (IBM, Tokyo, Japan).

#### 2.9.3. Microbiome Analysis

Microbiome analysis were performed by free software R ver 4.12 with microbiome and physeq package [39,40].

### 2.10. Ethics

Approval for the study was obtained from the Ethical Committee of Tsurumi University School of Dental Medicine (Approval Number: 1882) and followed the Consolidated Standards of Reporting Trials (CONSORT). Written informed consent was obtained from all of the patients included in the study. The consent was obtained from those over 20 years of age directly and from the parents of those under 20 years of age.

## 3. Results

### 3.1. Baseline Characteristics of the Patients Paticiapted in This Study

The baseline characteristics of the subjects participated in this study were shown in Table S1. No statistically significant differences were observed in demographic and clinical parameters between fluoride application group and tooth surface disinfection group.

### 3.2. Monitoring the Effect of Treatment by Salvaru Levels of Mutans Streptococci

**Figure 1:**
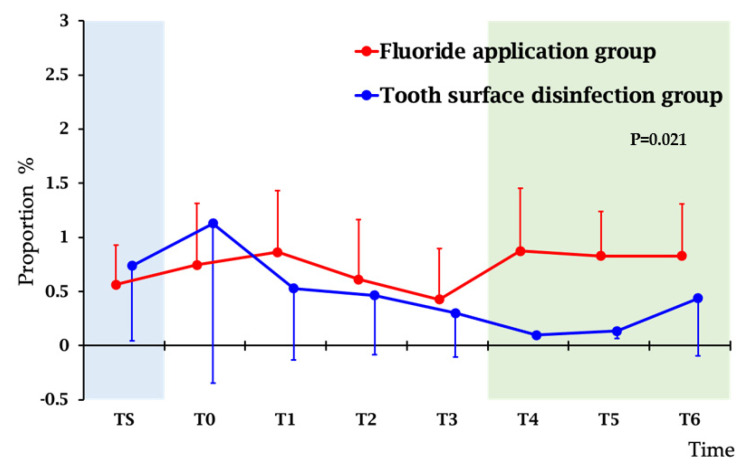
Changes of salivary levels of proportion of mutans streptococci in total streptococci during study periods. *p*-values were calculated by mixed effect model. Changes of cariogenic bacteria were all statistically significant. The results of mixed effect models were shown in Table S2. Time-points: TS, screening; T0, at the start of study (baseline); T1: after 1 month; T2: after 2 months; T3: after 3 months; T4: after 4 months; T5: after 5 months; T6: after 6 months.

To monitor the effect of treatment, salivary levels mutans streptococci were measured at screening (TS), baseline (T0), and monthly after the start of the treatment. Changes of proportion of mutans streptococci in total streptococci is shown in Figure 1. By the mixed effect modeling, tooth surface disinfection treatment significantly reduced both mutans streptococci and *Lactobacillus* (Figure S3 and Table S2).

### 3.3. Site Level Changes of the White Spot Regions Evalate by QLF

**Figure 2:**
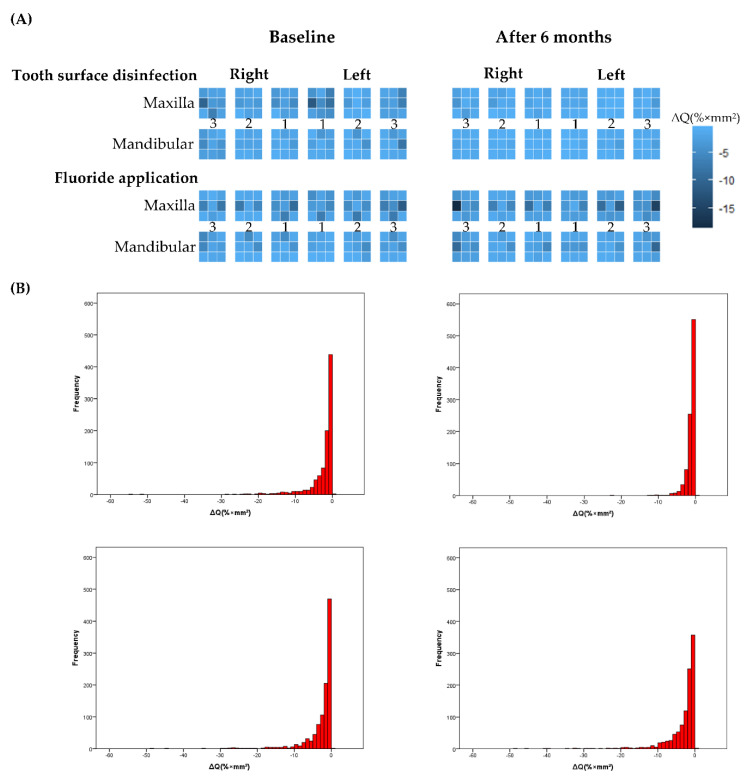
Changes of ΔQ value evaluated by QLF. (**A**) Heatmap by segmentation of nine areas in each tooth. (**B**) Histogram of white spot parameters.

Site level changes of white spot parameters (Area of white spot, ΔF, ΔF_max_, and ΔQ) were shown in Figure 2 and Figure S4. Site level changes of ΔQ value were illustrated as heatmap (Figure 2A). The values of ΔQ summarized by tooth type were illustrated as histogram (Figure 2B). By the observation of these Figures, tooth surface disinfection treatment effectively reduced WSLs. The heatmap and histogram of other parameters were presented in Figure S4. Summary statistics of each site at baseline and after 6 months were described in Table S3.

The results were shown in Table 1. Tooth surface disinfection significantly reduced all parameters obtained by QLF. The reductions were significantly different between tooth types.

When including clinical parameters supposed to be risk factors for dental caries in the model, only dental plaque levels evaluated by QLF were statistically significant. The results were shown in Table S4.

The graphs in upper row show tooth surface disinfection group and the in lower row show Fluoride application group. The left columns show baseline and the right column shows after six months. The changes of ΔQ value were illustrated by histogram by baseline and after six months. Tooth surface disinfection group clearly reduced the ΔQ value when compared with Fluoride application group. In heatmap, the lighter the color, the smaller the volume of WSLs. The four parameters (White spot area (mm^2^), ΔF (%), ΔF_max_ (%), ΔQ (% × mm^2^)) were evaluated, and this figure shows the change in volume (ΔQ (% × mm^2^) as a representative. The changes of other parameters are shown in the Figure S4.

For all parameters evaluated by QLF, Tooth surface disinfection treatment were significantly reduced the WSLs when Fluoride application used as reference. The reductions were varied between tooth type.

### 3.4. Microbiome Analysis before and after Treatment

#### 3.4.1. Sequence Data

From 44 samples from the 22 subjects, 2,116,945 reads (minimum, 20,573; maximum, 77,467) passed quality control. From these reads, sequences were clustered into 14 phyla, 30 classes, 52 orders, 85 families, 170 genera, and 550 species. The prevalence and abundances of all 550 species are visualized using a heatmap in Figure S5.

The summary statistics of the alpha diversity indices are shown in Table S5. The rarefaction curve is presented in Figure S6.

#### 3.4.2. Oral-Microbiome Structure

**Figure 3:**
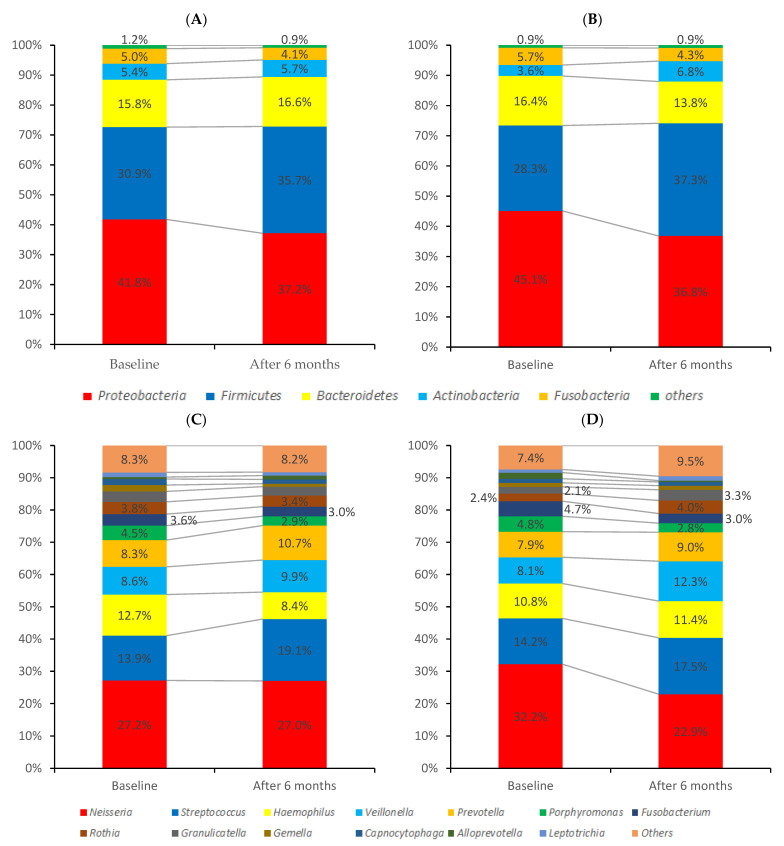
Oral microbiome structure before and after treatment. Data are presented separately phylum, and genus level. (**A**) Tooth surface disinfection group at phylum level. (**B**) Fluoride application group at phylum level. (**C**) Tooth surface disinfection group at genius level. (**D**) Fluoride application group at genus level.

Figure 3 shows the relative abundance of detected bacteria. Data are presented separately on the (A) phylum and (B) genus levels. The taxon prevalence of baseline and after treatments were shown in Figure S7. The prevalence of each species is plotted against their abundance. Highly prevalent phyla were Firmicutes and *Actinobacteria*. The core line plot and core heatmap are shown in Figure S8 and Figure S9.

#### 3.4.3. Effect of Tooth Surface Disinfection Treatment on Oral Microbiome

Species which has shown more than 0.1% changes are listed in Table 2. Some of the etiological bacteria for pneumonia were significantly reduced by the tooth surface disinfection treatment.

Effect on oral disease pathogenic bacteria were also shown in Table S6. The treatment effects were evaluated by mixed effect modeling. The results were shown in Table S7. Tooth surface disinfection treatment significantly reduced mutans streptococci.

#### 3.4.4. Correlation of White Spot and Oral Microbiome

**Figure 4:**
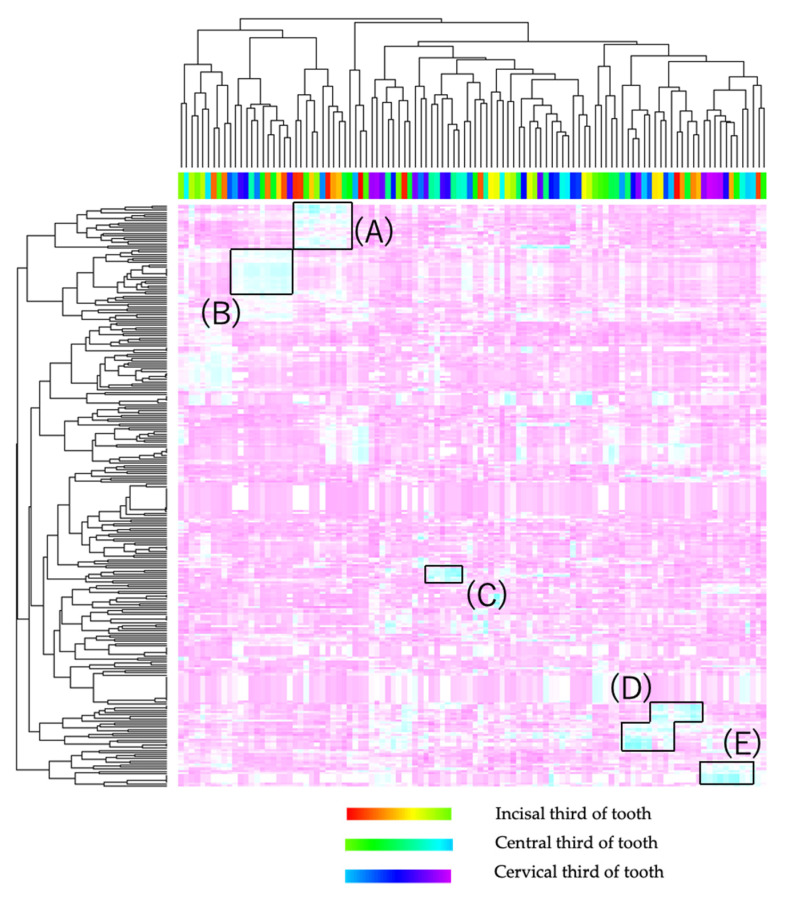
Correlation heatmap of ΔQ value and species. The order of tooth surface in legend is maxillary to mandibular, right to left, distal to mesial. Detailed correlations are shown in the Table S8. Sky blue area indicate positive correlations. Some of the sky-blue area contains site specific species: *Aggregatibacter*, *Alloprevotella*, *Anaerococcus*, *Bifidobacterium*, *Porphyromonas*, *Prevotella*, *Treponemma*, *Vagococcus*, *Veillonella*, and *Streptococci*. (**A**–**E**): Cluster of highly positive correlation between WSLs and species.

Correlation of oral white spot evaluate QLF and oral microbiome was illustrated as correlation heatmap. The results of ΔQ value and oral microbiome were shown in Figure 4. Positive correlations were illustrated as sky blue. Incisor area had positive correlations with *Actinomyces* (in Figure 4A). In contrast, cervical area had positive correlations with *Aggregatibacter*, *Alloprevotella*, *Anaerococcus*, *Bifidobacterium* (in Figure 4B), *Porphyromonas*, *Prevotella* (in Figure 4C), *Treponemma*, *Vagococcus* and *Veillonella* (in Figure 4E). In addition to, *Streptococci* was distributed throughout the tooth surface (in Figure 4D). Correlation heatmap by other QLF values were shown in Figure S10.

## 4. Discussion

Previous reports investigated to examine the optimum protocols for decreasing the levels of mutans streptococci to control dental caries. However, few clinical studies have been performed to examine the feasibility of applying those protocols in patients treated with fixed orthodontic appliances [18,19,41,42].

CHX is one of the most popular and well-studied antimicrobial agents used in the oral cavity [15,16]. Moreover, CHX has been shown to have a safety profile. However, reversible adverse effects, such as impaired taste sensation [43], tooth staining [41], and occasional mucus membrane irritation [43], have been associated with prolonged use of CHX application. Therefore, we used 0.20% CHX gel and applied it carefully so as to not leak CHX gel for oral mucosa. No patients had shown these side effects.

Our present report had shown that tooth surface disinfection treatment effectively reduced or eliminated mutans streptococci. As shown in Figure 1, we confirmed of the effect of tooth surface disinfection for the reduction of mutans streptococci throughout the six months of the study period. Our result of significant reduction in mutans streptococci levels is similar to the results of previous studies in which CHX gel or varnish were used [18,19,41,42].

Patients under orthodontic treatment develop significantly more WSLs than non-orthodontic patients. In addition, the progression of dental caries was faster in patients with fixed orthodontic appliances and its incidence was higher in canines and premolars [44,45]. WSLs can become noticeable around the brackets within one month after bracket placement, although the formation of dental cavity usually takes at least six months. WSLs are superficial and have the potential to remineralize. Therefore, early diagnosis enables the clinician to implement minimally invasive treatments with the use of remineralization therapies.

With respect to the diagnosis of dental caries, the conventional methods (visual and radiographic examination) present low sensitivity for quantifying the changes in mineral content as result of demineralization and remineralization [46]. To overcome this limitation, QLF system has been studied as alternative method to quantify differences between sound and demineralized enamel, showing a correlation with TMR ranging from 0.62 to 0.84 for demineralized and from 0.66 to 0.84 for remineralized enamel [47,48,49]. The QLF system consists of an image analysis which can calculate the percentage of fluorescence loss of the selected enamel area based on the amount of mineral loss during the analysis period. A strong reproducibility of the method and validated the QLF system was presented [50,51]. This system capable of recording a minor area of demineralization during orthodontic treatment with more than 5% fluorescence loss detection. It is not possible to be detected in a visual or clinical examination. Therefore, QLF seems to be a useful method to quantify demineralization and monitor the treatment of WSLs. Moreover, earlier studies have shown that QLF can disclose remineralization of WSLs after an interval of only six weeks [52].

There are many approaches used to prevent enamel demineralization during orthodontic treatment [53,54] and the post-orthodontic stage [55,56] and to obtain remineralization on demineralized surfaces [57]. Some of them include the use and application of fluorine-containing agents [13,14], casein phosphopeptide containing agents [17], and antimicrobial agents [15,16]. The fact that CHX is an agent frequently applied for chemical biofilm control in patients with fixed orthodontic appliances indirectly leads to possible effects on the remineralization of active WSLs. Our results showed that two applications of 0.2% CHX gel in addition to PMTC and fluoride application increased the QLF parameters at the patient level, tooth level, and site level (Table 1).

Conventional statistical models tend to overlook multilevel structures and disturbed independency among observations, which leads to type I errors and potential misinterpretations [37]. Multilevel modeling is one of the techniques of mixed effects analyses and has been employed for dental caries data or periodontal data. Previous studies have specified the risk factors that are associated with the progression of dental caries conditions at the patient, tooth, and site levels [21,58]. One of the novel findings in this study is that the improvement of changes of white spots evaluated by QLF were dependent on the tooth type.

The oral cavity harbors more than 700 bacterial species, constituting one of the most diverse bacterial communities in the human body [59]. Oral cavity comprises complex structures of hard and soft tissue, such as the teeth, tongue, gingiva, and palate. Unique variations in the oral microbiome structure are observed according to the different surface properties. In this study, we revealed the changes of the oral microbiome and compared before and after tooth surface disinfection treatment at the bacterial phylum, genus, and species level by using mixed effect models.

Comparing the composition of oral microbiome at baseline (T0) and after 6 months (T6), the changes were not statistically significant at the phylum and genus level (Figure 3). However, at the bacterial species level, more than 0.1% changes were observed in some species (Table 2).

In the view of bacterial replacement, the concentration of *Streptococcus salivarius*, known as a probiotic bacterium, increased after tooth surface disinfection treatment when compared with the fluoride application group. It shows that bacterial permutation from oral pathogenic bacteria (especially mutans streptococci) to probiotics bacteria had occurred by the effect of tooth surface disinfection treatment.

Correlation heatmap shown in Figure 4 indicate that Anaerobic bacteria had a positive correlation with ΔQ value in cervical area. *Aggregatibacter*, *Alloprevotella*, *Anaerococcus*, *Bifidobacterium*, *Porphyromonas*, *Prevotella*, *Treponemma*, *Vagococcus*, and *Veillonella* had positive correlation with ΔQ value in cervical area (Figure 4B,C,E). This may be due to the fact that the cervical area is close to the gingival sulcus, which is an anaerobic environment. On the other hand, some aerobic bacterial species including *Actinomyces* distributed in areas away from the cervical area (in Figure 4A). Additionally, Streptococci was distributed all over the tooth surface (in Figure 4D).

The main limitation of this study was that the monitoring period was six months. A longer period with more monitoring time-points may be required to better detect any changes in oral pathogenic bacteria levels. Even though the sample size was enough at the tooth level, it was relatively small at the patient level.

However, disinfection of tooth surfaces during orthodontic treatment may reduce the risk of dental caries and systematic disease by reducing or eliminating the pathogenic bacteria from oral cavity.

## 5. Conclusions

Tooth surface disinfection treatment, in addition to PMTC and fluoride application. more effectively reduce the WSLs during fixed orthodontic treatment.

## Figures and Tables

**Table 1:** Results of mixed effect modeling for the changes of QLF parameters before and after treatment.

			White Spot Area (mm^2^)		ΔF (%)		ΔF_max_ (%)		ΔQ(% × mm^2^)	
			Coefficient (95% CI)	*p*-Value	Coefficient (95% CI)	*p*-Value	Coefficient (95% CI)	*p*-Value	Coefficient (95% CI)	*p*-Value
Fixed effect										
Intercept			0.375 (0.314–0.435)	<0.001	−2.195 (−2.234–−2.156)	<0.001	−2.662 (−2.606–−2.718)	<0.001	−1.063 (−1.357–−0.769)	<0.001
Treatment	Tooth surface disinfection		−0.142 (−0.211–−0.074)	<0.001	0.076 (0.027–0.125)	0.003	0.223 (0.155–0.292)	<0.001	0.636 (0.260–1.012)	0.001
	Fluoride application		Reference		Reference		Reference		Reference	
Time	After 6 months		−0.043 (−0.061–−0.025)	<0.001	0.038 (0.027–0.048)	<0.001	0.106 (0.084–0.128)	<0.001	0.345 (0.295–0.396)	<0.001
	Baseline		Reference		Reference		Reference		Reference	
Tooth type	Maxillary	1	0.054 (−0.002–0.11)	0.059	0.094 (0.069–0.120)	<0.001	0.116 (0.079–0.153)	<0.001	0.005 (0.193–0.202)	0.963
		2	−0.024 (−0.082–0.034)	0.413	0.056 (0.030–0.082)	<0.001	0.090 (0.052–0.128)	<0.001	0.394 (0.190–0.598)	<0.001
		3	0.082 (0.031–0.134)	0.002	0.014 (0.010–0.037)	0.249	−0.002 (0.036–0.032)	0.898	−0.123 (0.305–0.059)	0.186
	Mandibular	1	−0.097 (−0.149–−0.045)	<0.001	0.061 (0.038–0.085)	<0.001	0.103 (0.069–0.138)	<0.001	0.566 (0.381–0.750)	<0.001
		2	−0.092 (−0.147–−0.037)	0.001	0.055 (0.030–0.080)	<0.001	0.082 (0.045–0.118)	<0.001	0.476 (0.283–0.670)	<0.001
		3	Reference		Reference		Reference		Reference	
Random effect										
Level1 (Subject)			0.005 (0.002–0.012)	0.012	−0.003 (−0.006–−0.002)	0.004	−0.006 (−0.012–−0.003)	0.004	−0.184 (−0.363–−0.094)	0.004
Level2 (Tooth)			0.006 (0.004–0.010)	<0.001	−0.001 (−0.002–−0.001)	0.001	<0.001	-	−0.093 (−0.138–−0.063)	<0.001
Level3 (Ordination)			0.041 (0.036–0.048)	<0.001	−0.009 (−0.011–−0.008)	<0.001	−0.021 (−0.028–−0.017)	<0.001	−0.535 (−0.598–−0.478)	<0.001
Fitness index										
AICC			2959.631		−2033.758		2904.679		11,802.447	
BIC			2991.11		−2002.287		2936.152		11,833.918	
Distribution			Normal, Link: Identity		*Γ*, Link: log		*Γ*, Link: log		*Γ*, Link: log	

**Table 2:** Species level effect if the tooth surface disinfection treatment on oral microbiome. (**A**) Tooth surface disinfection group at species level. (**B**) Fluoride application group at species level.

(**A**)							
**Species (0.1% or More Reduction)**	**Baseline** **(%)**	**After 6 Months** **(%)**	**Difference** **(%)**	**Species (0.1% or More Increase)**	**Baseline** **(%)**	**After 6 Months** **(%)**	**Difference** **(%)**
*Haemophilus parainfluenzae group*	11.18	7.16	−4.02	*Streptococcus sinensis group*	1.91	4.59	2.68
*Neisseria_uc*	2.07	0.07	−1.99	*Streptococcus parasanguinis group*	0.93	2.83	1.90
*Porphyromonas pasteri*	4.24	2.61	−1.62	*Streptococcus salivarius group*	1.07	2.66	1.60
*Streptococcus pneumoniae group*	8.18	7.13	−1.04	*Neisseria subflava*	11.56	13.01	1.45
*Gemella haemolysans group*	1.75	0.74	−1.02	*Veillonella rogosae*	1.96	3.16	1.20
*Veillonella parvula group*	1.40	0.86	−0.55	*Prevotella nanceiensis group*	0.43	1.35	0.91
*Fusobacterium nucleatum group*	1.28	0.77	−0.51	*Veillonella atypica*	0.72	1.39	0.67
*Granulicatella adiacens group*	3.04	2.57	−0.47	*Prevotella histicola*	0.31	0.80	0.49
*Prevotella denticola*	0.51	0.09	−0.42	*Prevotella melaninogenica*	4.56	5.05	0.49
*Streptococcus gordonii group*	0.68	0.36	−0.32	*Haemophilus pittmaniae*	0.05	0.48	0.43
*Haemophilus sputorum*	0.76	0.49	−0.27	*Actinomyces graevenitzii*	0.11	0.50	0.39
*Neisseria elongata group*	0.68	0.42	−0.26	*PAC001346_s*	0.26	0.63	0.38
*Haemophilus haemolyticus*	0.30	0.04	−0.26	*Neisseria oralis*	0.17	0.53	0.36
*Capnocytophaga leadbetteri*	0.58	0.34	−0.24	*Neisseria sicca group*	3.14	3.46	0.32
*Abiotrophia defectiva*	0.49	0.28	−0.20	*FJ976422_s*	0.12	0.44	0.31
*Capnocytophaga sputigena*	0.51	0.33	−0.18	*Prevotella pallens*	0.53	0.80	0.27
*KV831974_s group*	2.48	2.29	−0.18	*Streptococcus_uc*	0.25	0.50	0.25
*Rothia dentocariosa*	0.42	0.24	−0.18	*Lautropia mirabilis*	0.27	0.52	0.25
*KI272869_s*	0.32	0.17	−0.14	*Prevotella jejuni*	0.14	0.37	0.23
*AM420132_s*	0.17	0.03	−0.13	*Prevotella aurantiaca*	0.00	0.20	0.20
*JQ463704_s*	0.32	0.20	−0.12	*Actinomyces odontolyticus*	0.09	0.29	0.20
*Prevotella loescheii*	0.17	0.06	−0.12	*PAC001345_s*	0.24	0.44	0.20
*Haemophilus_uc*	0.17	0.05	−0.11	*Streptococcus peroris group*	0.37	0.54	0.17
*Dialister invisus*	0.25	0.13	−0.11	*LT608321_s*	0.04	0.19	0.16
*Haemophilus influenzae group*	0.18	0.07	−0.11	*Peptostreptococcus massiliae group*	0.06	0.20	0.14
*Prevotella oris*	0.19	0.08	−0.10	*Atopobium parvulum*	0.27	0.39	0.12
*EU681966_s*	0.10	0.00	−0.10	*JN713562_g_uc*	0.00	0.12	0.12
				*FJ976402_s*	0.04	0.16	0.12
				*CAGY_s*	0.02	0.13	0.11
				*Megasphaera micronuciformis*	0.41	0.51	0.10
(**B**)							
**Species (0.1% or More Reduction)**	**Baseline** **(%)**	**After 6 Months** **(%)**	**Difference** **(%)**	**Species (0.1% or More Increase)**	**Baseline** **(%)**	**After 6 Months** **(%)**	**Difference** **(%)**
*Neisseria perflava*	14.06	5.21	−8.85	*Prevotella veroralis*	0.02	0.14	0.11
*Neisseria subflava*	13.80	9.67	−4.13	*JQ463704_s*	0.19	0.31	0.12
*Porphyromonas pasteri*	4.46	2.65	−1.80	*Streptococcus gordonii group*	0.23	0.35	0.12
*Fusobacterium periodonticum group*	3.66	1.86	−1.80	*Streptococcus salivarius group*	1.75	1.88	0.13
*FJ976422_s*	1.02	0.03	−1.00	*Campylobacter gracilis*	0.08	0.21	0.13
*Prevotella nanceiensis group*	1.39	0.51	−0.88	*Corynebacterium matruchotii*	0.06	0.20	0.14
*LT608321_s*	0.60	0.10	−0.50	*JVLH_s*	0.04	0.19	0.15
*Neisseria elongata group*	1.07	0.58	−0.50	*Dialister invisus*	0.08	0.24	0.16
*Peptostreptococcus massiliae group*	0.46	0.06	−0.41	*Streptococcus peroris group*	0.57	0.73	0.17
*Prevotella aurantiaca*	0.32	0.08	−0.24	*Streptococcus_uc*	0.33	0.51	0.18
*Prevotella pallens*	0.56	0.34	−0.23	*Streptococcus sanguinis group*	0.77	0.95	0.18
*Haemophilus influenzae group*	0.37	0.18	−0.20	*KE952139_s*	0.17	0.36	0.19
*Haemophilus sputorum*	0.40	0.23	−0.17	*Prevotella jejuni*	0.34	0.54	0.20
*KI272869_s*	0.29	0.12	−0.16	*Rothia aeria*	0.39	0.60	0.21
*Aggregatibacter aphrophilus*	0.48	0.37	−0.11	*PAC001350_s*	0.13	0.35	0.22
*Capnocytophaga gingivalis*	0.15	0.04	−0.11	*Veillonella rogosae*	2.79	3.01	0.22
*JF239777_s*	0.13	0.03	−0.10	*Lautropia mirabilis*	0.41	0.66	0.25
*Haemophilus pittmaniae*	0.45	0.35	−0.10	*Veillonella atypica*	0.68	0.95	0.28
*JX294356_s*	0.11	0.00	−0.10	*Prevotella salivae*	0.16	0.47	0.30
				*Atopobium parvulum*	0.22	0.53	0.32
				*Prevotella_uc*	0.08	0.41	0.32
				*Abiotrophia defectiva*	0.15	0.49	0.33
				*Rothia dentocariosa*	0.15	0.59	0.44
				*KV831974_s group*	1.41	1.88	0.47
				*Rothia mucilaginosa group*	0.44	0.96	0.52
				*Streptococcus parasanguinis group*	1.73	2.27	0.54
				*Prevotella melaninogenica*	3.85	4.43	0.59
				*Prevotella histicola*	0.17	0.76	0.59
				*Streptococcus pneumoniae group*	7.12	8.01	0.89
				*Streptococcus sinensis group*	1.54	2.47	0.93
				*Haemophilus parainfluenzae group*	9.14	10.25	1.12
				*Granulicatella adiacens group*	1.86	3.03	1.17
				*Veillonella parvula group*	0.71	1.97	1.26
				*Neisseria_uc*	0.09	1.57	1.48
				*Veillonella dispar*	3.81	6.18	2.37
				*Neisseria sicca group*	2.78	5.42	2.64

## Data Availability

All of the clinical data and microbiome data are available in the Supplementary Materials.

## Supplementary Materials

The following supporting information can be downloaded at: https://www.mdpi.com/article/10.3390/jcm11030854/s1. Figure S1: Flowchart showing recruitment of patients, allocation and dropout; Figure S2: Each site was divided as shown in the conceptual diagram above; Figure S3: Monitoring the effect of treatment by saliva levels of cariogenic bacteria; Figure S4: Heatmap and Histogram of white spot parameters evaluated by QLF; Figure S5: Heatmap of 550 species detected in this study; Figure S6: Rare fraction curves of 44 samples; Figure S7: Taxon prevalence; Figure S8: Core line plot of the oral microbiome; Figure S9: Core heatmap of oral microbiome; Figure S10: Correlation heatmap by other QLF values; Table S1: Statistics issued during randomization; Table S2: The results of mixed effect models in monitoring of cariogenic bacteria; Table S3: Summary statistics of the site level QLF parameters; Table S4: Results of changes of QLF parameters by mixed effect modeling with risk factors supposed to be dental caries; Table S5: The summary statistics of the alpha diversity indices; Table S6: The treatment effects on oral disease pathogenic bacteria; Table S7: The results of treatment effects on oral disease pathogenic bacteria evaluated by mixed effect modeling; Table S8: Detailed correspondence table of allocation numbers and bacterial species in correlation heatmap; File S1: Model Specification; File S2: All of the data analyzed in this study.
