# Supplementary material for: Effects of the Dental Caries Preventive Procedure on the White Spot Lesions during Orthodontic Treatment—An Open Label Randomized Controlled Trial
Source: J Clin Med. 2022 Feb 6;11(3):854. doi: 10.3390/jcm11030854 (PMC8836958; doi:10.3390/jcm11030854)
Supplement: Supplementary file 1 [file jcm-11-00854-s001.zip › file S1.docx]

●Model 1

Multilevel random intercept model for the changes in White spot values between the baseline and after 6 months

Site, Tooth and Patient are indexed by *i*, *j*, *k*, respectively.

-L1:

$$\left( \Delta White spot values \right)_{ijk}=\pi_{0jk}+r_{ijk}$$

-L2:

$$\pi_{0jk}=\beta_{00k}+\sum_{m=2}^{6} \beta_{01k}^{\left( n \right)}\left( Tooth type indexed by n \right)_{\mathrm{jk}}+r_{0jk}$$

$\pi_{1jk}=\beta_{10k}$

$$\pi_{2jk}^{\left( m \right)}=\beta_{20k}^{\left( m \right)}$$

-L3:

$$\beta_{00k}=\gamma_{000}+\gamma_{001}^{\left( 2 \right)}{(treatment)}_{k}+\gamma_{002}^{\left( 2 \right)}{(time)}_{k}+u_{000}+X$$

$$\beta_{01k}^{\left( n \right)}=\gamma_{010}^{\left( n \right)}$$

$\beta_{10k}=\gamma_{100}$

$\beta_{20k}^{\left( m \right)}=\gamma_{200}^{\left( m \right)}$,

where $e_{ijk}\sim\kappa\left( 0, \sigma_{e}^{2} \right), r_{0jk}\sim\kappa\left( 0, \sigma_{r}^{2} \right), u_{00k}\sim\kappa\left( 0, \sigma_{u}^{2} \right)$

Data Structure: Patient, Tooth, Site

Random effect

Patient level: Intercept; Random effect covariance: variance component

Tooth level: Intercept; Random effect covariance: variance component

●Model 2

Multilevel random intercept model for the changes in Oral pathogenic bacteria between the baseline and after 6 months

Site, Tooth and Patient are indexed by *i*, *j*, *k*, respectively.

-L1:

$$\left( \Delta Oral pathogenic bacteria \right)_{ijk}=\pi_{0jk}+r_{ijk}$$

-L2:

$$\pi_{0jk}=\beta_{00k}+{\beta_{01k}^{\left( 2 \right)}\left( \mathrm{treatment} \right)}_{\mathrm{jk}}+{\beta_{02k}^{\left( 2 \right)}\left( \mathrm{time} \right)}_{\mathrm{jk}}+r_{0jk}$$

$\pi_{1jk}=\beta_{10k}$

$$\pi_{2jk}^{\left( m \right)}=\beta_{20k}^{\left( m \right)}$$

where $e_{ijk}\sim\kappa\left( 0, \sigma_{e}^{2} \right), r_{0jk}\sim\kappa\left( 0, \sigma_{r}^{2} \right)$

Data Structure: Patient, Tooth

Random effect

Patient level: Intercept; Random effect covariance: variance component

Tooth level: Intercept; Random effect covariance: variance component

π_0_: Site level intercept

π_1,2..._: Site level coefficient

*i*: Site level ID

ε: Site level random effect

β_0_: Tooth level intercept

β_1,2..._: Tooth level coefficient

*j*: Tooth level ID

r : Tooth level random effect

γ_0_: Subject level intercept

γ_1,2..._: Subject level coefficient

*κ*: Subject level ID

μ: Subject level random effect

X is none, *mutans streptococci*, *Lactobacillus*.
