# Supplementary material for: Effects of the Dental Caries Preventive Procedure on the White Spot Lesions during Orthodontic Treatment—An Open Label Randomized Controlled Trial
Source: J Clin Med. 2022 Feb 6;11(3):854. doi: 10.3390/jcm11030854 (PMC8836958; doi:10.3390/jcm11030854)
Supplement: Supplementary file 1 [file Supplemental.pdf › Supplemental.pdf]

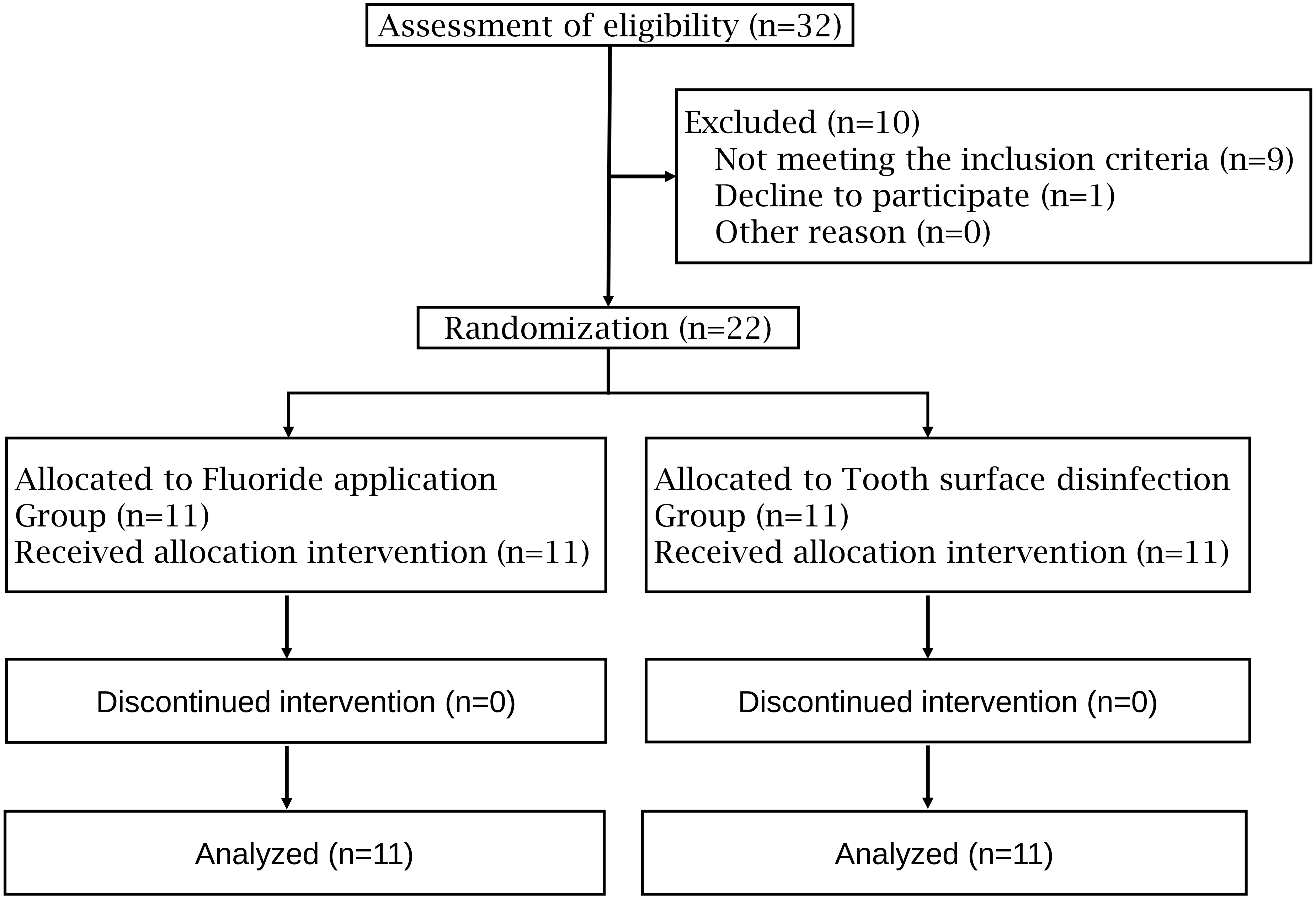

Figure S1. Flowchart showing recruitment of patients, allocation and dropout

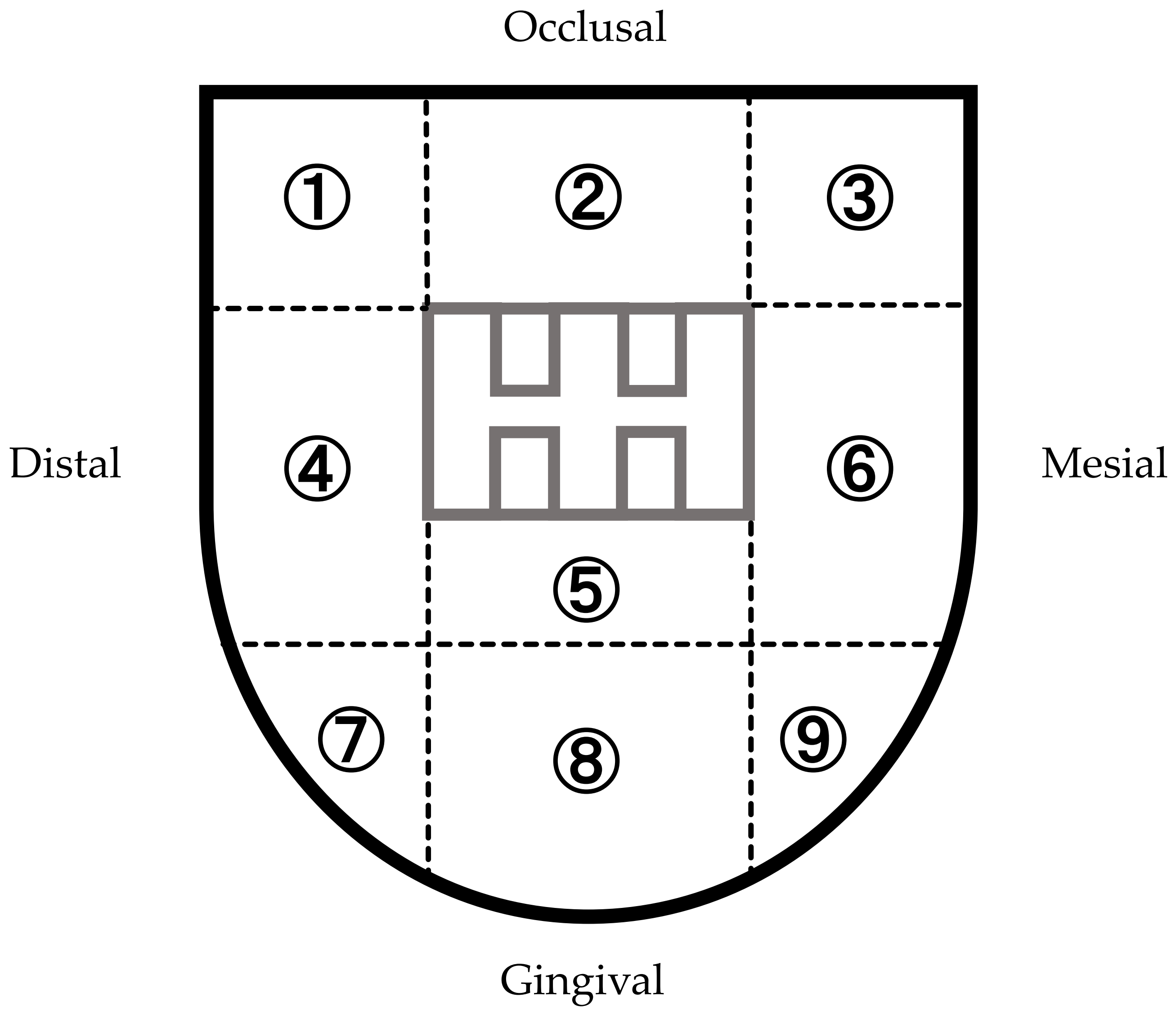

Figure S2. The site of tooth surface used in this study.

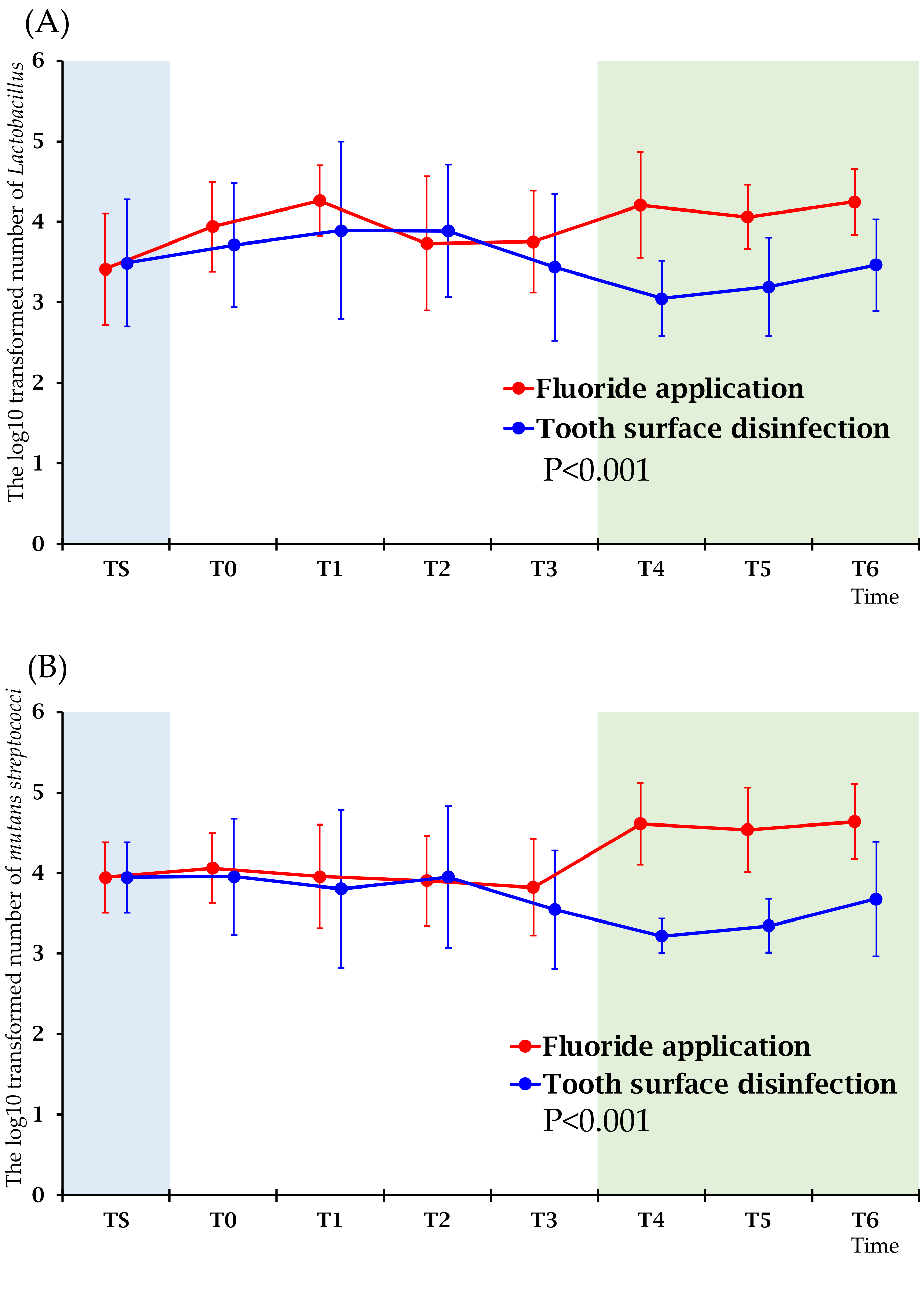

Figure S3. Monitoring the effect of treatment by saliva levels of cariogenic bacteria

(A) The log10 transformed number of *Lactobacillus*
(B) The log10 transformed number of *mutans streptococci*
P-value was observed by mixed effect model.
The mixed effect model was shown in Table S2.

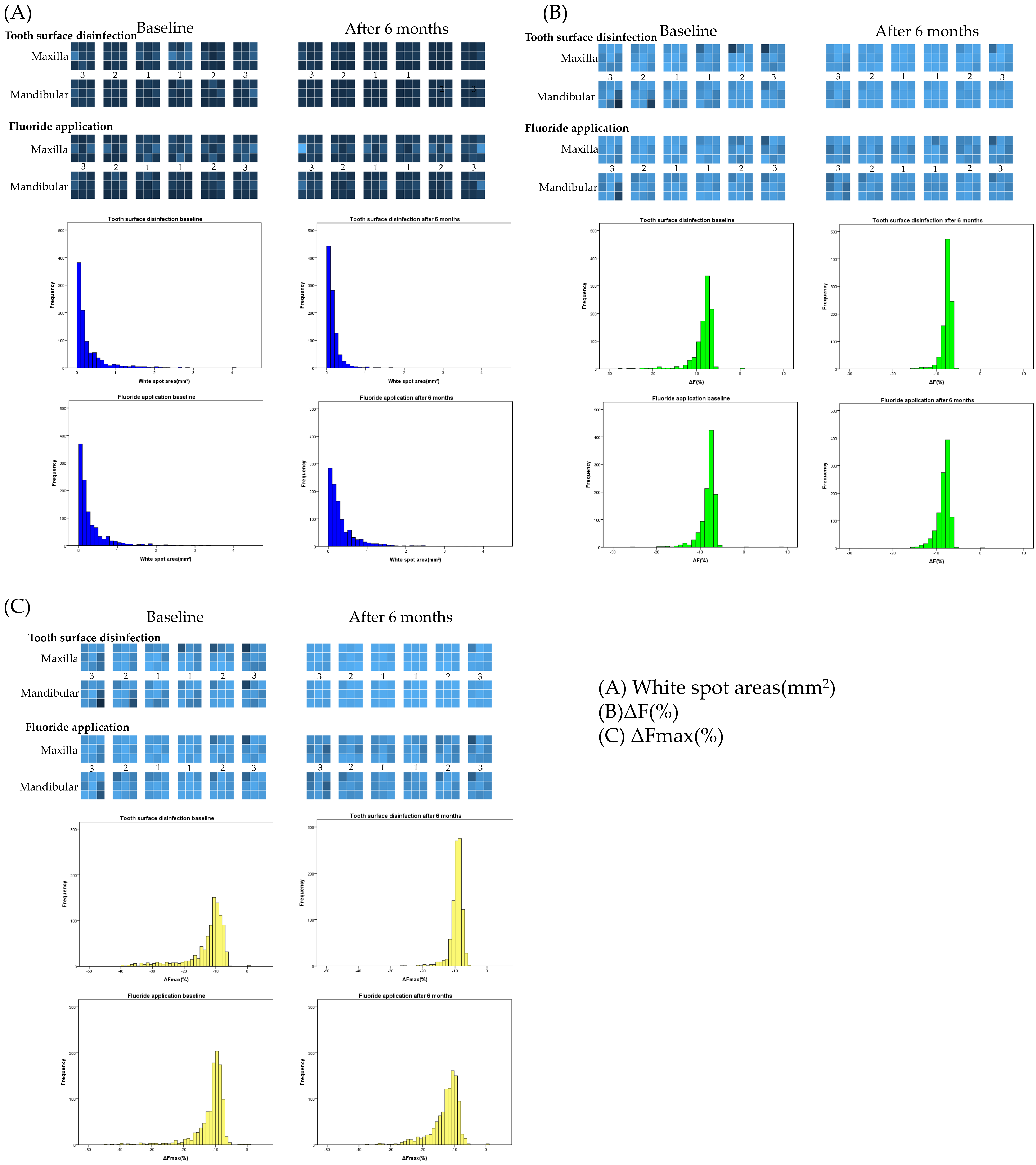

Figure S4. Heatmap and Histogram of white spot parameters evaluated by QLF

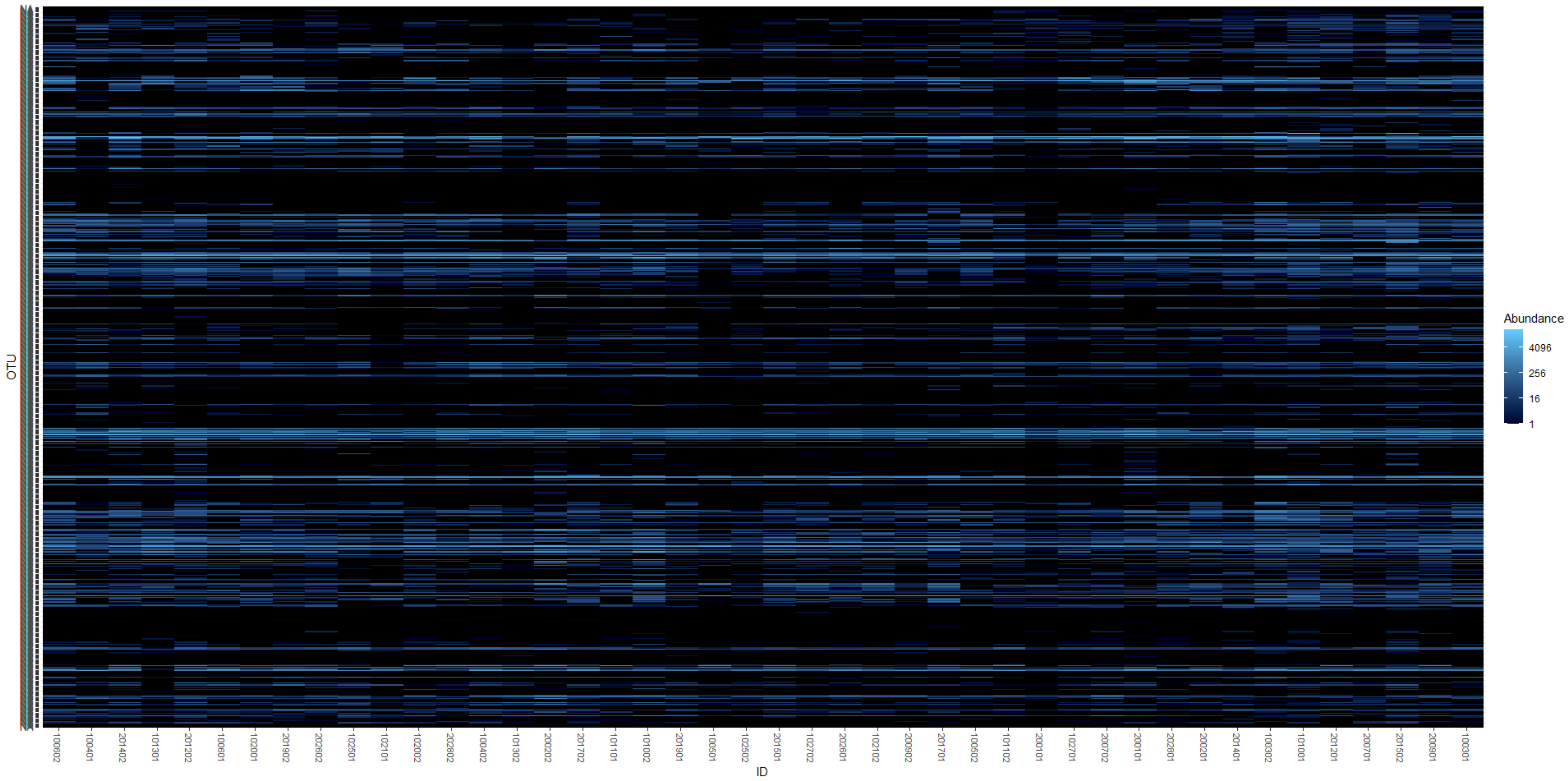

Figure S5. Heatmap of 550 species detected in this study

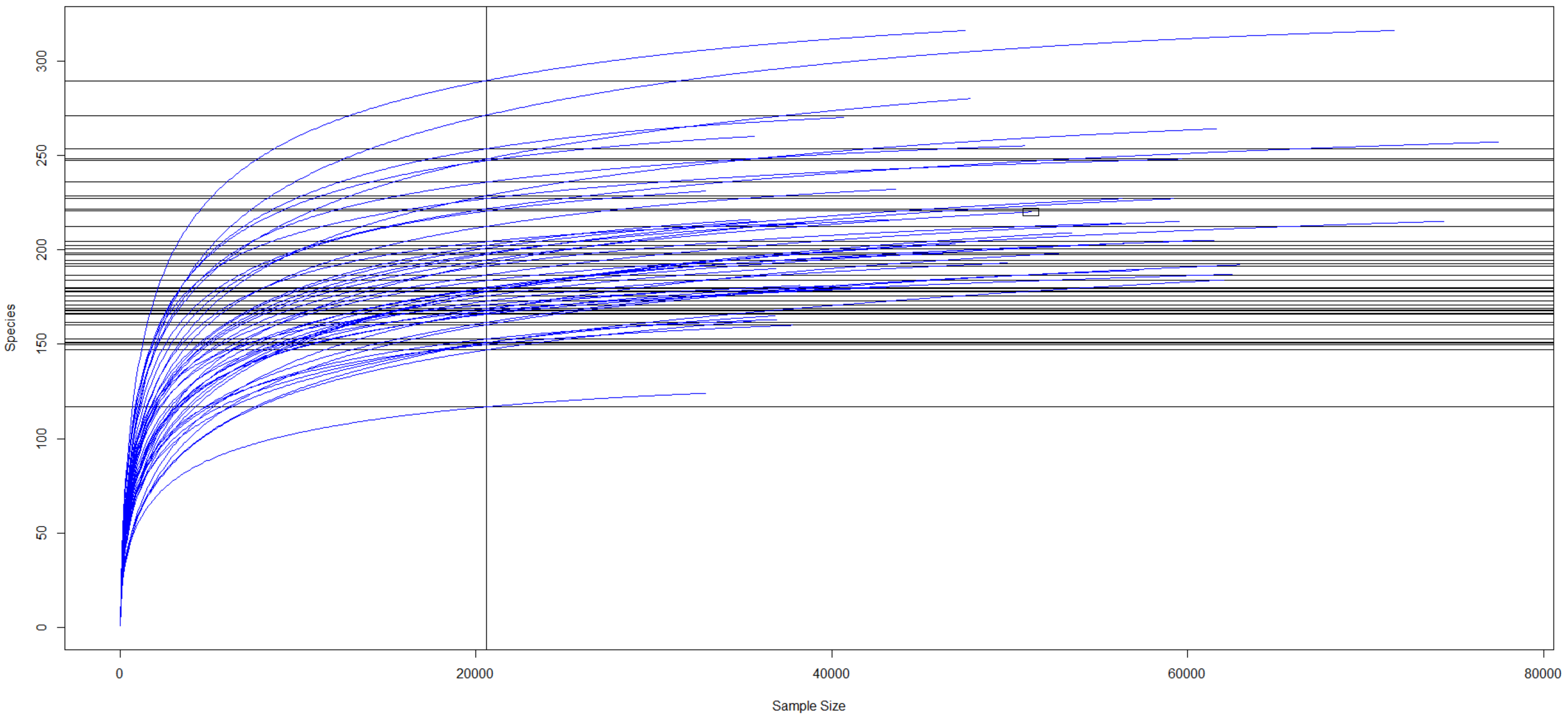

Figure S6. Rare fraction curves of 44 samples

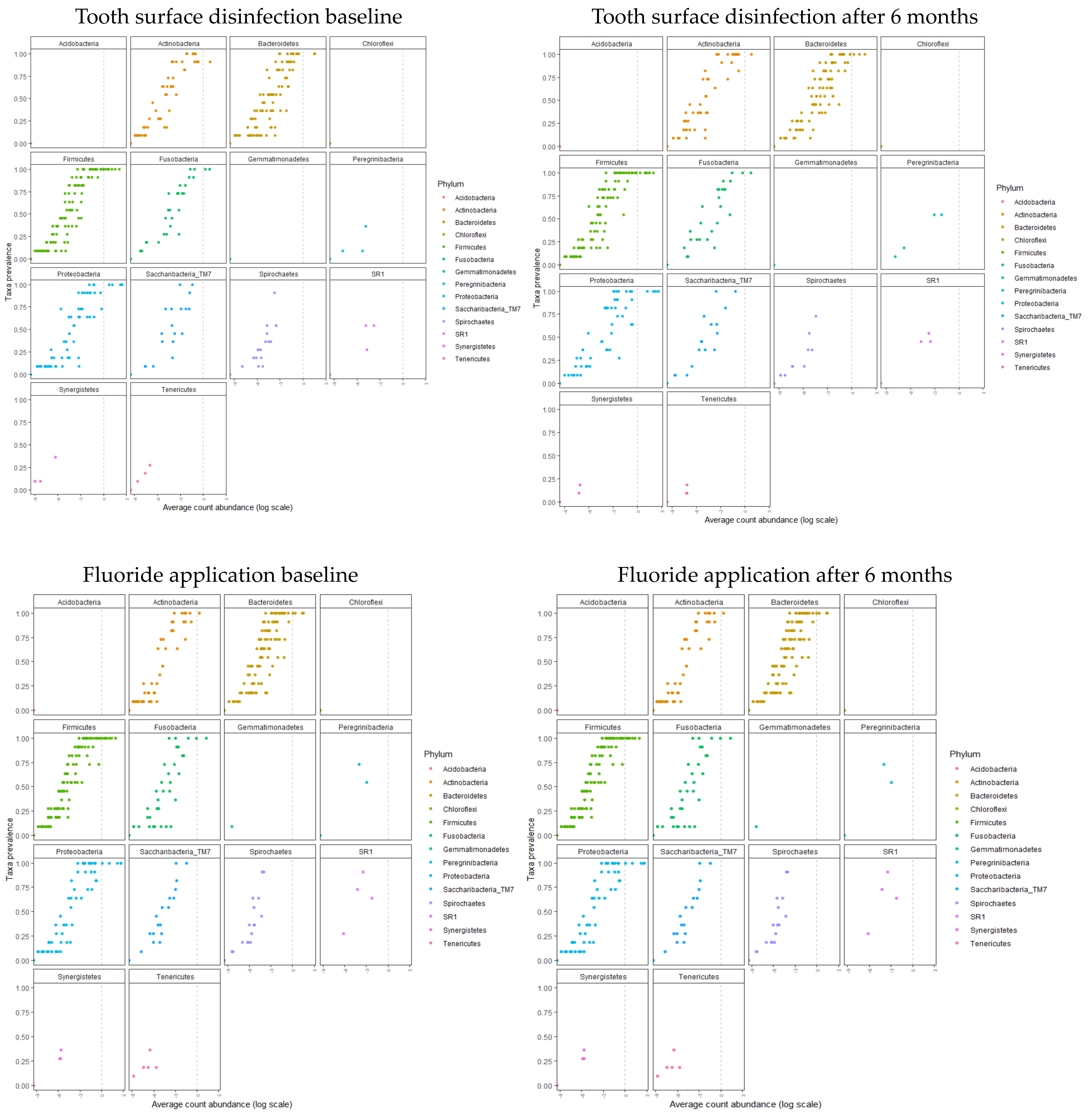

Figure S7. Taxon prevalence

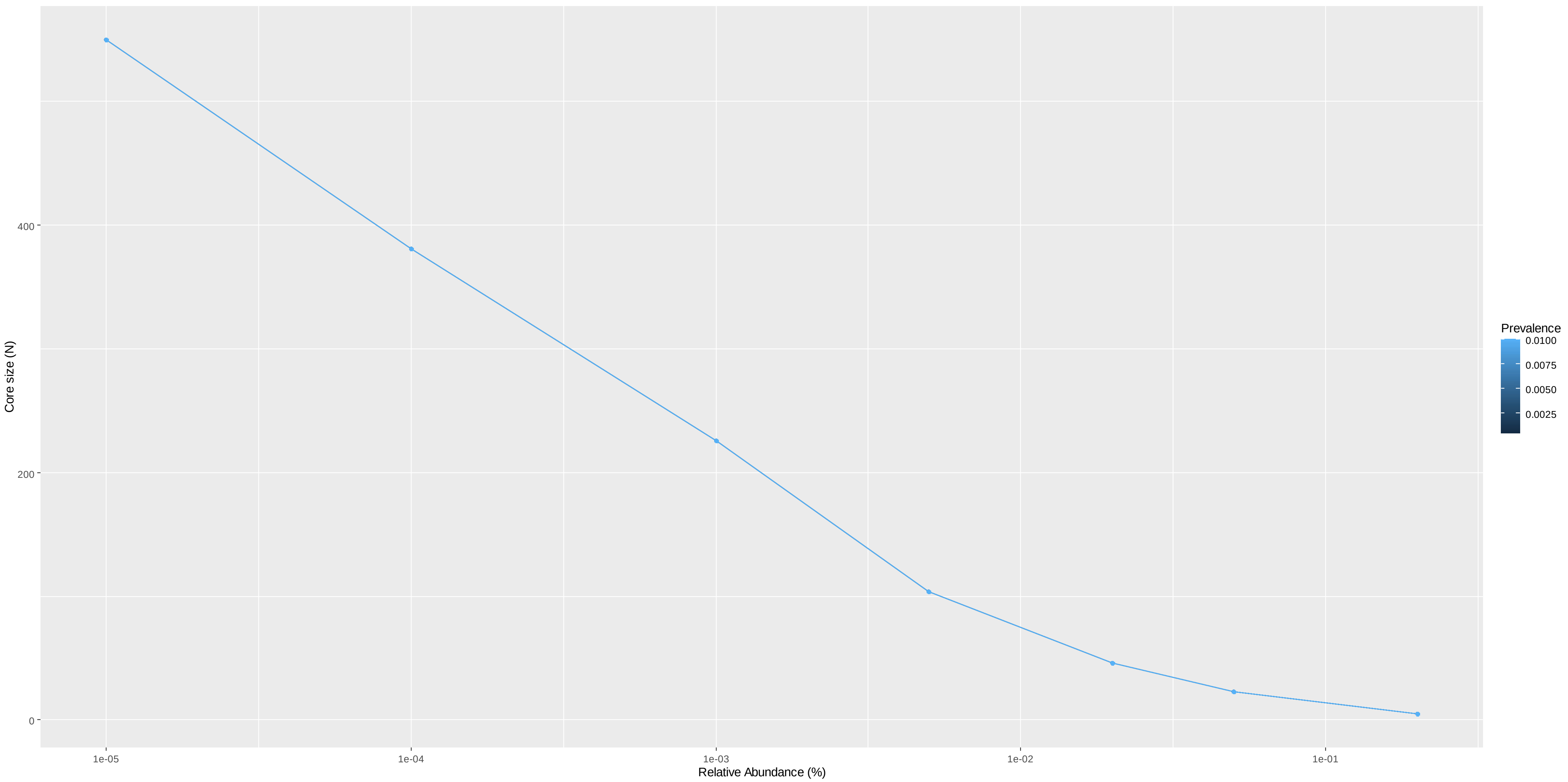

Figure S8: Core line plot

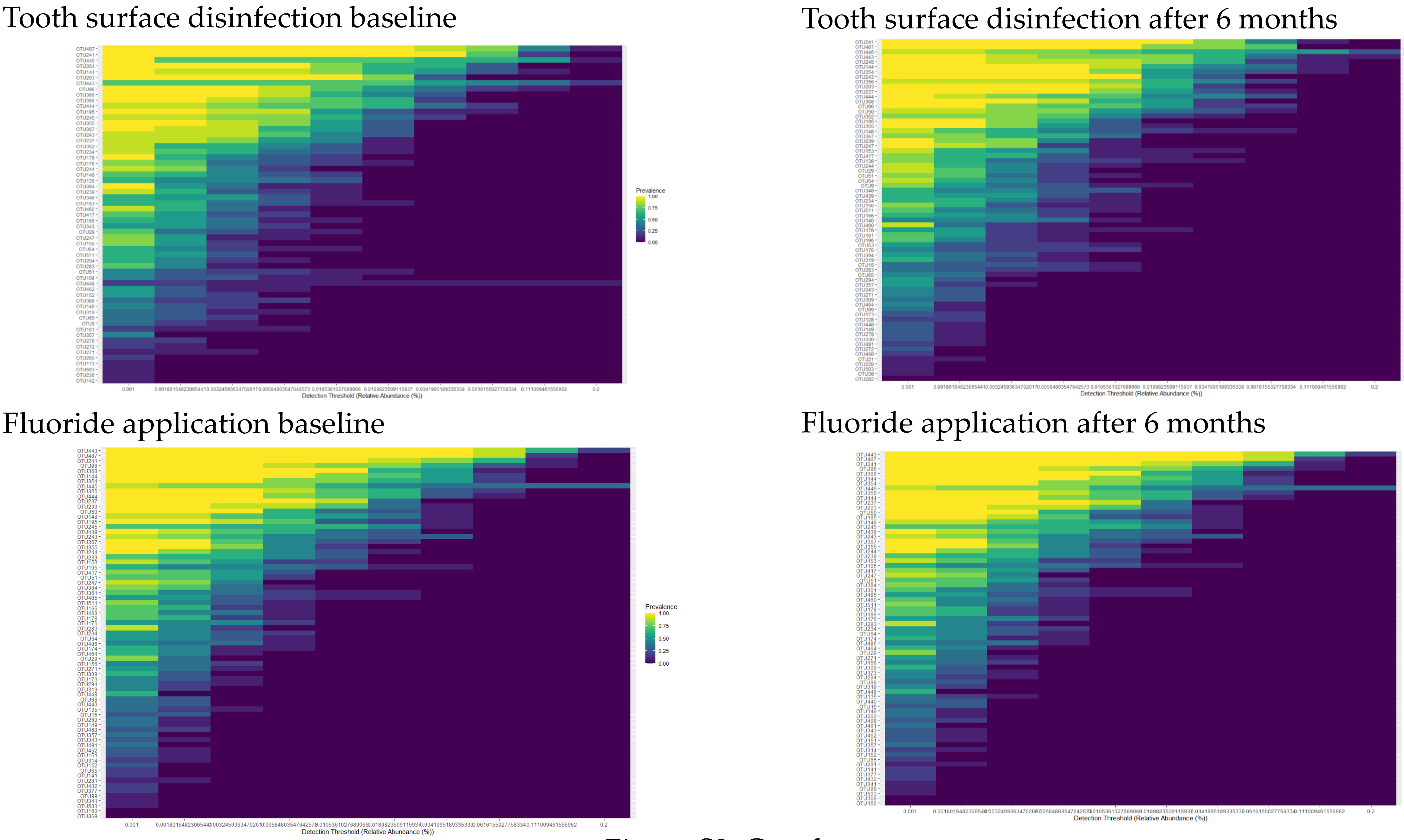

Figure S9: Core heatmaps

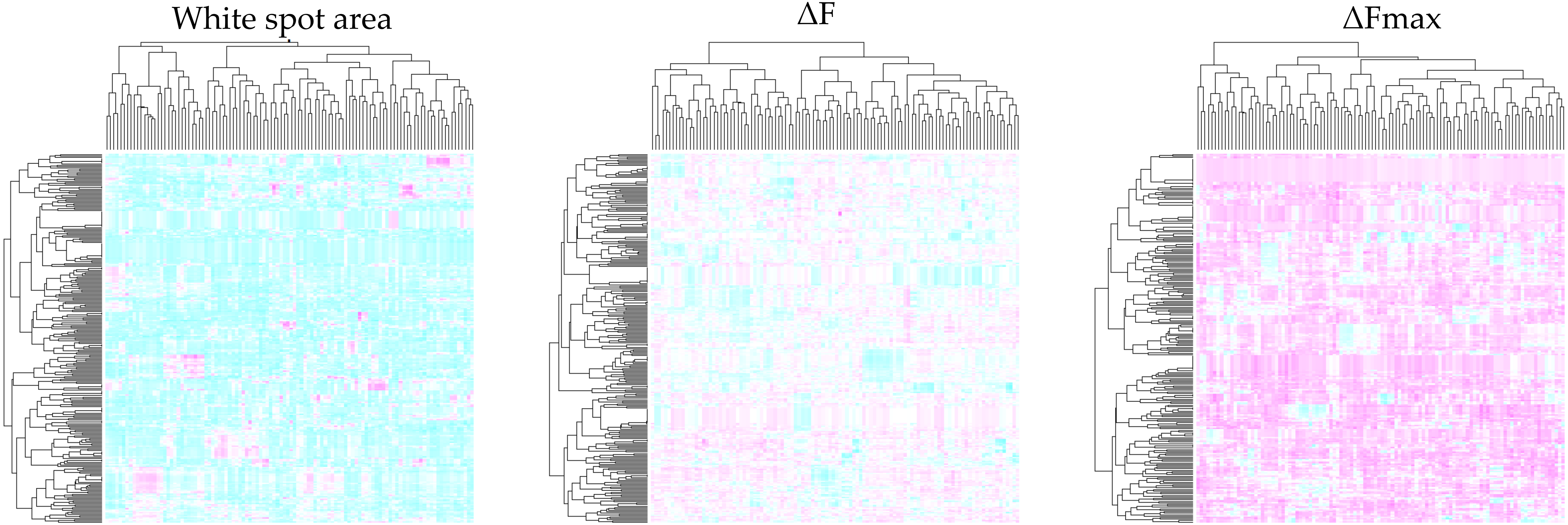

Figure S10. Correlation heatmap by other QLF values

Table S1. Statistics issued during randomization

		Allocation1(n=11)	Allocation2(n=11)	P-value	Statistical test
DMF index		5.0 [2.0, 7.0]	3.0 [1.8, 10.0]	0.39	b
Five-min stimulated saliva volume (ml)		6.1±2.3	5.9±2.5	0.864	a
The proportion of *mutans streptococci* (%)		0.45[0.28, 1.00]	0.35[0.10, 1.63]	0.782	b
Mutans streptococci (log $_{10}$ (cfu)/ml saliva)	Mean +/-SD	3.9±0.5	3.7±1.2	0.618	a
	Median (25th-75th)	3.9[3.7, 4.0]	4.0[3.4,4.5]	0.782	b
Lactobacillus (log $_{10}$ (cfu)/ml saliva)	Mean +/-SD	2.7±1.8	2.7±2.1	0.974	a
	Median (25th-75th)	3.7[0.0,4.0]	3.9[0.0,4.1]	0.454	b
Orthodontic Diagnosis					
Crowding		4(28.6%)	4(28.6%)	1	c
Maxillary protrusion		3(21.4%)	4(28.6%)		
Mandibular protrusion		6(42.9%)	5(35.7%)		
Open bite		1(7.1%)	0(0%)		
Bimaxillary protrusion		0(0.0%)	1(7.1%)		
Treatment duration					
Less than one year		9(81.8%)	10(90.9%)	0.628	b
Over one year		2(18.2%)	1(9.1%)		
Lip closure failure					
Included		3(27.3%)	3(27.3%)	1	b
Not included		8(72.7%)	8(72.7%)		
Disease index					
0		0(0%)	0(0%)	0.918	b
1		1(9.1%)	1(9.1%)		
2		2(18.1%)	2(18.1%)		
3		1(9.1%)	0(0%)		
4		7(63.6%)	8(72.7%)		
Risk factor					
0		0(0%)	0(0%)	0.577	b
1		0(0%)	2(18.1%)		
2		6(54.5%)	5(45.5%)		
3		5(45.5%)	2(18.1%)		
4		0(0%)	1(9.1%)		
5		0(0%)	1(9.1%)		
6-8		0(0%)	0(0%)		
Defensive factor					
0		0(0%)	0(0%)	0.704	b
1		4(36.4%)	5(45.5%)		
2		7(63.6%)	6(54.5%)		
3-8		0(0%)	0(0%)		
Risk level					
Low and Medium		0(0%)	0(0%)	1	b
High		10(90.9%)	10(90.9%)		
Extreme		1(9.1%)	1(9.1%)		

Table S2. The results of mixed effect models in monitoring of cariogenic bacteria

			The log10 transformed number of *Lactobacillus*		Proportion of *mutans streptococci*		The log10 transformed number of *mutans streptococci*	
			Coefficient (95% CI)	P-value	Coefficient (95% CI)	P-value	Coefficient (95% CI)	P-value
Fixed effect	Intercept		1.393(1.311-1.475)	＜0.001	0.822(0.573-1.072)	＜0.001	1.439(1.37-1.508)	＜0.001
	Treatment	Tooth surface disinfection	-0.162(-0.251--0.073)	＜0.001	-0.334(-0.617--0.052)	0.021	-0.152(-0.232--0.072)	＜0.001
		Fluoride application	Reference		Reference		Reference	
	Time		0(-0.009-0.01)	0.935	-0.019(-0.049-0.01)	0.198	0.001(-0.007-0.009)	0.821
Random effect	Intercept		0.008(0.003-0.02)	0.026	0.088(0.036-0.22)	0.031	0.007(0.03-0.003)	0.016
Fitness index		AICC	-64.649		296.972		-122.876	
		BIC	-37.374		324.248		-95.601	
Link function			F log		Normal ,Identity		F log	

Table S3. Summary statistics of the site level QLF parameters.

				White spot area(mm^2)																																			
				Right																		Left																	
				3						2						1						1						2						3					
				Distal		Central		Medial		Distal		Central		Medial		Distal		Central		Medial		Medial		Central		Distal		Medial		Central		Distal		Medial		Central		Distal	
				Mean	Median	Mean	Median	Mean	Median	Mean	Median	Mean	Median	Mean	Median	Mean	Median	Mean	Median	Mean	Median	Mean	Median	Mean	Median	Mean	Median	Mean	Median	Mean	Median	Mean	Median	Mean	Median	Mean	Median	Mean	Median
Tooth surface disinfection	Baseline	Maxilla	Edge	[illegible]	[illegible]	[illegible]	[illegible]	[illegible]	[illegible]	[illegible]	[illegible]	[illegible]	[illegible]	[illegible]	[illegible]	[illegible]	[illegible]	[illegible]	[illegible]	[illegible]	[illegible]	[illegible]	[illegible]	[illegible]	[illegible]	[illegible]	[illegible]	[illegible]	[illegible]	[illegible]	[illegible]	[illegible]	[illegible]	[illegible]	[illegible]	[illegible]	[illegible]	[illegible]	[illegible]
			Central	[illegible]	[illegible]	[illegible]	[illegible]	[illegible]	[illegible]	[illegible]	[illegible]	[illegible]	[illegible]	[illegible]	[illegible]	[illegible]	[illegible]	[illegible]	[illegible]	[illegible]	[illegible]	[illegible]	[illegible]	[illegible]	[illegible]	[illegible]	[illegible]	[illegible]	[illegible]	[illegible]	[illegible]	[illegible]	[illegible]	[illegible]	[illegible]	[illegible]	[illegible]	[illegible]	[illegible]
			Cervical	[illegible]	[illegible]	[illegible]	[illegible]	[illegible]	[illegible]	[illegible]	[illegible]	[illegible]	[illegible]	[illegible]	[illegible]	[illegible]	[illegible]	[illegible]	[illegible]	[illegible]	[illegible]	[illegible]	[illegible]	[illegible]	[illegible]	[illegible]	[illegible]	[illegible]	[illegible]	[illegible]	[illegible]	[illegible]	[illegible]	[illegible]	[illegible]	[illegible]	[illegible]	[illegible]	[illegible]
		Mandibular	Edge	[illegible]	[illegible]	[illegible]	[illegible]	[illegible]	[illegible]	[illegible]	[illegible]	[illegible]	[illegible]	[illegible]	[illegible]	[illegible]	[illegible]	[illegible]	[illegible]	[illegible]	[illegible]	[illegible]	[illegible]	[illegible]	[illegible]	[illegible]	[illegible]	[illegible]	[illegible]	[illegible]	[illegible]	[illegible]	[illegible]	[illegible]	[illegible]	[illegible]	[illegible]	[illegible]	[illegible]
			Central	[illegible]	[illegible]	[illegible]	[illegible]	[illegible]	[illegible]	[illegible]	[illegible]	[illegible]	[illegible]	[illegible]	[illegible]	[illegible]	[illegible]	[illegible]	[illegible]	[illegible]	[illegible]	[illegible]	[illegible]	[illegible]	[illegible]	[illegible]	[illegible]	[illegible]	[illegible]	[illegible]	[illegible]	[illegible]	[illegible]	[illegible]	[illegible]	[illegible]	[illegible]	[illegible]	[illegible]
			Cervical	[illegible]	[illegible]	[illegible]	[illegible]	[illegible]	[illegible]	[illegible]	[illegible]	[illegible]	[illegible]	[illegible]	[illegible]	[illegible]	[illegible]	[illegible]	[illegible]	[illegible]	[illegible]	[illegible]	[illegible]	[illegible]	[illegible]	[illegible]	[illegible]	[illegible]	[illegible]	[illegible]	[illegible]	[illegible]	[illegible]	[illegible]	[illegible]	[illegible]	[illegible]	[illegible]	[illegible]
	After 6 months	Maxilla	Edge	[illegible]	[illegible]	[illegible]	[illegible]	[illegible]	[illegible]	[illegible]	[illegible]	[illegible]	[illegible]	[illegible]	[illegible]	[illegible]	[illegible]	[illegible]	[illegible]	[illegible]	[illegible]	[illegible]	[illegible]	[illegible]	[illegible]	[illegible]	[illegible]	[illegible]	[illegible]	[illegible]	[illegible]	[illegible]	[illegible]	[illegible]	[illegible]	[illegible]	[illegible]	[illegible]	[illegible]
			Central	[illegible]	[illegible]	[illegible]	[illegible]	[illegible]	[illegible]	[illegible]	[illegible]	[illegible]	[illegible]	[illegible]	[illegible]	[illegible]	[illegible]	[illegible]	[illegible]	[illegible]	[illegible]	[illegible]	[illegible]	[illegible]	[illegible]	[illegible]	[illegible]	[illegible]	[illegible]	[illegible]	[illegible]	[illegible]	[illegible]	[illegible]	[illegible]	[illegible]	[illegible]	[illegible]	[illegible]
			Cervical	[illegible]	[illegible]	[illegible]	[illegible]	[illegible]	[illegible]	[illegible]	[illegible]	[illegible]	[illegible]	[illegible]	[illegible]	[illegible]	[illegible]	[illegible]	[illegible]	[illegible]	[illegible]	[illegible]	[illegible]	[illegible]	[illegible]	[illegible]	[illegible]	[illegible]	[illegible]	[illegible]	[illegible]	[illegible]	[illegible]	[illegible]	[illegible]	[illegible]	[illegible]	[illegible]	[illegible]
		Mandibular	Edge	[illegible]	[illegible]	[illegible]	[illegible]	[illegible]	[illegible]	[illegible]	[illegible]	[illegible]	[illegible]	[illegible]	[illegible]	[illegible]	[illegible]	[illegible]	[illegible]	[illegible]	[illegible]	[illegible]	[illegible]	[illegible]	[illegible]	[illegible]	[illegible]	[illegible]	[illegible]	[illegible]	[illegible]	[illegible]	[illegible]	[illegible]	[illegible]	[illegible]	[illegible]	[illegible]	[illegible]
			Central	[illegible]	[illegible]	[illegible]	[illegible]	[illegible]	[illegible]	[illegible]	[illegible]	[illegible]	[illegible]	[illegible]	[illegible]	[illegible]	[illegible]	[illegible]	[illegible]	[illegible]	[illegible]	[illegible]	[illegible]	[illegible]	[illegible]	[illegible]	[illegible]	[illegible]	[illegible]	[illegible]	[illegible]	[illegible]	[illegible]	[illegible]	[illegible]	[illegible]	[illegible]	[illegible]	[illegible]
			Cervical	[illegible]	[illegible]	[illegible]	[illegible]	[illegible]	[illegible]	[illegible]	[illegible]	[illegible]	[illegible]	[illegible]	[illegible]	[illegible]	[illegible]	[illegible]	[illegible]	[illegible]	[illegible]	[illegible]	[illegible]	[illegible]	[illegible]	[illegible]	[illegible]	[illegible]	[illegible]	[illegible]	[illegible]	[illegible]	[illegible]	[illegible]	[illegible]	[illegible]	[illegible]	[illegible]	[illegible]
Fluoride application	Baseline	Maxilla	Edge	[illegible]	[illegible]	[illegible]	[illegible]	[illegible]	[illegible]	[illegible]	[illegible]	[illegible]	[illegible]	[illegible]	[illegible]	[illegible]	[illegible]	[illegible]	[illegible]	[illegible]	[illegible]	[illegible]	[illegible]	[illegible]	[illegible]	[illegible]	[illegible]	[illegible]	[illegible]	[illegible]	[illegible]	[illegible]	[illegible]	[illegible]	[illegible]	[illegible]	[illegible]	[illegible]	[illegible]
			Central	[illegible]	[illegible]	[illegible]	[illegible]	[illegible]	[illegible]	[illegible]	[illegible]	[illegible]	[illegible]	[illegible]	[illegible]	[illegible]	[illegible]	[illegible]	[illegible]	[illegible]	[illegible]	[illegible]	[illegible]	[illegible]	[illegible]	[illegible]	[illegible]	[illegible]	[illegible]	[illegible]	[illegible]	[illegible]	[illegible]	[illegible]	[illegible]	[illegible]	[illegible]	[illegible]	[illegible]
			Cervical	[illegible]	[illegible]	[illegible]	[illegible]	[illegible]	[illegible]	[illegible]	[illegible]	[illegible]	[illegible]	[illegible]	[illegible]	[illegible]	[illegible]	[illegible]	[illegible]	[illegible]	[illegible]	[illegible]	[illegible]	[illegible]	[illegible]	[illegible]	[illegible]	[illegible]	[illegible]	[illegible]	[illegible]	[illegible]	[illegible]	[illegible]	[illegible]	[illegible]	[illegible]	[illegible]	[illegible]
		Mandibular	Edge	[illegible]	[illegible]	[illegible]	[illegible]	[illegible]	[illegible]	[illegible]	[illegible]	[illegible]	[illegible]	[illegible]	[illegible]	[illegible]	[illegible]	[illegible]	[illegible]	[illegible]	[illegible]	[illegible]	[illegible]	[illegible]	[illegible]	[illegible]	[illegible]	[illegible]	[illegible]	[illegible]	[illegible]	[illegible]	[illegible]	[illegible]	[illegible]	[illegible]	[illegible]	[illegible]	[illegible]
			Central	[illegible]	[illegible]	[illegible]	[illegible]	[illegible]	[illegible]	[illegible]	[illegible]	[illegible]	[illegible]	[illegible]	[illegible]	[illegible]	[illegible]	[illegible]	[illegible]	[illegible]	[illegible]	[illegible]	[illegible]	[illegible]	[illegible]	[illegible]	[illegible]	[illegible]	[illegible]	[illegible]	[illegible]	[illegible]	[illegible]	[illegible]	[illegible]	[illegible]	[illegible]	[illegible]	[illegible]
			Cervical	[illegible]	[illegible]	[illegible]	[illegible]	[illegible]	[illegible]	[illegible]	[illegible]	[illegible]	[illegible]	[illegible]	[illegible]	[illegible]	[illegible]	[illegible]	[illegible]	[illegible]	[illegible]	[illegible]	[illegible]	[illegible]	[illegible]	[illegible]	[illegible]	[illegible]	[illegible]	[illegible]	[illegible]	[illegible]	[illegible]	[illegible]	[illegible]	[illegible]	[illegible]	[illegible]	[illegible]
	After 6 months	Maxilla	Edge	[illegible]	[illegible]	[illegible]	[illegible]	[illegible]	[illegible]	[illegible]	[illegible]	[illegible]	[illegible]	[illegible]	[illegible]	[illegible]	[illegible]	[illegible]	[illegible]	[illegible]	[illegible]	[illegible]	[illegible]	[illegible]	[illegible]	[illegible]	[illegible]	[illegible]	[illegible]	[illegible]	[illegible]	[illegible]	[illegible]	[illegible]	[illegible]	[illegible]	[illegible]	[illegible]	[illegible]
			Central	[illegible]	[illegible]	[illegible]	[illegible]	[illegible]	[illegible]	[illegible]	[illegible]	[illegible]	[illegible]	[illegible]	[illegible]	[illegible]	[illegible]	[illegible]	[illegible]	[illegible]	[illegible]	[illegible]	[illegible]	[illegible]	[illegible]	[illegible]	[illegible]	[illegible]	[illegible]	[illegible]	[illegible]	[illegible]	[illegible]	[illegible]	[illegible]	[illegible]	[illegible]	[illegible]	[illegible]
			Cervical	[illegible]	[illegible]	[illegible]	[illegible]	[illegible]	[illegible]	[illegible]	[illegible]	[illegible]	[illegible]	[illegible]	[illegible]	[illegible]	[illegible]	[illegible]	[illegible]	[illegible]	[illegible]	[illegible]	[illegible]	[illegible]	[illegible]	[illegible]	[illegible]	[illegible]	[illegible]	[illegible]	[illegible]	[illegible]	[illegible]	[illegible]	[illegible]	[illegible]	[illegible]	[illegible]	[illegible]
		Mandibular	Edge	[illegible]	[illegible]	[illegible]	[illegible]	[illegible]	[illegible]	[illegible]	[illegible]	[illegible]	[illegible]	[illegible]	[illegible]	[illegible]	[illegible]	[illegible]	[illegible]	[illegible]	[illegible]	[illegible]	[illegible]	[illegible]	[illegible]	[illegible]	[illegible]	[illegible]	[illegible]	[illegible]	[illegible]	[illegible]	[illegible]	[illegible]	[illegible]	[illegible]	[illegible]	[illegible]	[illegible]
			Central	[illegible]	[illegible]	[illegible]	[illegible]	[illegible]	[illegible]	[illegible]	[illegible]	[illegible]	[illegible]	[illegible]	[illegible]	[illegible]	[illegible]	[illegible]	[illegible]	[illegible]	[illegible]	[illegible]	[illegible]	[illegible]	[illegible]	[illegible]	[illegible]	[illegible]	[illegible]	[illegible]	[illegible]	[illegible]	[illegible]	[illegible]	[illegible]	[illegible]	[illegible]	[illegible]	[illegible]
			Cervical	[illegible]	[illegible]	[illegible]	[illegible]	[illegible]	[illegible]	[illegible]	[illegible]	[illegible]	[illegible]	[illegible]	[illegible]	[illegible]	[illegible]	[illegible]	[illegible]	[illegible]	[illegible]	[illegible]	[illegible]	[illegible]	[illegible]	[illegible]	[illegible]	[illegible]	[illegible]	[illegible]	[illegible]	[illegible]	[illegible]	[illegible]	[illegible]	[illegible]	[illegible]	[illegible]	[illegible]

				ΔF(%)																																			
				Right																		Left																	
				3						2						1						1						2						3					
				Distal		Central		Medial		Distal		Central		Medial		Distal		Central		Medial		Medial		Central		Distal		Medial		Central		Distal		Medial		Central		Distal	
				Mean	Median	Mean	Median	Mean	Median	Mean	Median	Mean	Median	Mean	Median	Mean	Median	Mean	Median	Mean	Median	Mean	Median	Mean	Median	Mean	Median	Mean	Median	Mean	Median	Mean	Median	Mean	Median	Mean	Median	Mean	Median
Tooth surface disinfection	Baseline	Maxilla	Edge	[illegible]	[illegible]	[illegible]	[illegible]	[illegible]	[illegible]	[illegible]	[illegible]	[illegible]	[illegible]	[illegible]	[illegible]	[illegible]	[illegible]	[illegible]	[illegible]	[illegible]	[illegible]	[illegible]	[illegible]	[illegible]	[illegible]	[illegible]	[illegible]	[illegible]	[illegible]	[illegible]	[illegible]	[illegible]	[illegible]	[illegible]	[illegible]	[illegible]	[illegible]	[illegible]	[illegible]
			Central	[illegible]	[illegible]	[illegible]	[illegible]	[illegible]	[illegible]	[illegible]	[illegible]	[illegible]	[illegible]	[illegible]	[illegible]	[illegible]	[illegible]	[illegible]	[illegible]	[illegible]	[illegible]	[illegible]	[illegible]	[illegible]	[illegible]	[illegible]	[illegible]	[illegible]	[illegible]	[illegible]	[illegible]	[illegible]	[illegible]	[illegible]	[illegible]	[illegible]	[illegible]	[illegible]	[illegible]
			Cervical	[illegible]	[illegible]	[illegible]	[illegible]	[illegible]	[illegible]	[illegible]	[illegible]	[illegible]	[illegible]	[illegible]	[illegible]	[illegible]	[illegible]	[illegible]	[illegible]	[illegible]	[illegible]	[illegible]	[illegible]	[illegible]	[illegible]	[illegible]	[illegible]	[illegible]	[illegible]	[illegible]	[illegible]	[illegible]	[illegible]	[illegible]	[illegible]	[illegible]	[illegible]	[illegible]	[illegible]
		Mandibular	Edge	[illegible]	[illegible]	[illegible]	[illegible]	[illegible]	[illegible]	[illegible]	[illegible]	[illegible]	[illegible]	[illegible]	[illegible]	[illegible]	[illegible]	[illegible]	[illegible]	[illegible]	[illegible]	[illegible]	[illegible]	[illegible]	[illegible]	[illegible]	[illegible]	[illegible]	[illegible]	[illegible]	[illegible]	[illegible]	[illegible]	[illegible]	[illegible]	[illegible]	[illegible]	[illegible]	[illegible]
			Central	[illegible]	[illegible]	[illegible]	[illegible]	[illegible]	[illegible]	[illegible]	[illegible]	[illegible]	[illegible]	[illegible]	[illegible]	[illegible]	[illegible]	[illegible]	[illegible]	[illegible]	[illegible]	[illegible]	[illegible]	[illegible]	[illegible]	[illegible]	[illegible]	[illegible]	[illegible]	[illegible]	[illegible]	[illegible]	[illegible]	[illegible]	[illegible]	[illegible]	[illegible]	[illegible]	[illegible]
			Cervical	[illegible]	[illegible]	[illegible]	[illegible]	[illegible]	[illegible]	[illegible]	[illegible]	[illegible]	[illegible]	[illegible]	[illegible]	[illegible]	[illegible]	[illegible]	[illegible]	[illegible]	[illegible]	[illegible]	[illegible]	[illegible]	[illegible]	[illegible]	[illegible]	[illegible]	[illegible]	[illegible]	[illegible]	[illegible]	[illegible]	[illegible]	[illegible]	[illegible]	[illegible]	[illegible]	[illegible]
	After 6 months	Maxilla	Edge	[illegible]	[illegible]	[illegible]	[illegible]	[illegible]	[illegible]	[illegible]	[illegible]	[illegible]	[illegible]	[illegible]	[illegible]	[illegible]	[illegible]	[illegible]	[illegible]	[illegible]	[illegible]	[illegible]	[illegible]	[illegible]	[illegible]	[illegible]	[illegible]	[illegible]	[illegible]	[illegible]	[illegible]	[illegible]	[illegible]	[illegible]	[illegible]	[illegible]	[illegible]	[illegible]	[illegible]
			Central	[illegible]	[illegible]	[illegible]	[illegible]	[illegible]	[illegible]	[illegible]	[illegible]	[illegible]	[illegible]	[illegible]	[illegible]	[illegible]	[illegible]	[illegible]	[illegible]	[illegible]	[illegible]	[illegible]	[illegible]	[illegible]	[illegible]	[illegible]	[illegible]	[illegible]	[illegible]	[illegible]	[illegible]	[illegible]	[illegible]	[illegible]	[illegible]	[illegible]	[illegible]	[illegible]	[illegible]
			Cervical	[illegible]	[illegible]	[illegible]	[illegible]	[illegible]	[illegible]	[illegible]	[illegible]	[illegible]	[illegible]	[illegible]	[illegible]	[illegible]	[illegible]	[illegible]	[illegible]	[illegible]	[illegible]	[illegible]	[illegible]	[illegible]	[illegible]	[illegible]	[illegible]	[illegible]	[illegible]	[illegible]	[illegible]	[illegible]	[illegible]	[illegible]	[illegible]	[illegible]	[illegible]	[illegible]	[illegible]
		Mandibular	Edge	[illegible]	[illegible]	[illegible]	[illegible]	[illegible]	[illegible]	[illegible]	[illegible]	[illegible]	[illegible]	[illegible]	[illegible]	[illegible]	[illegible]	[illegible]	[illegible]	[illegible]	[illegible]	[illegible]	[illegible]	[illegible]	[illegible]	[illegible]	[illegible]	[illegible]	[illegible]	[illegible]	[illegible]	[illegible]	[illegible]	[illegible]	[illegible]	[illegible]	[illegible]	[illegible]	[illegible]
			Central	[illegible]	[illegible]	[illegible]	[illegible]	[illegible]	[illegible]	[illegible]	[illegible]	[illegible]	[illegible]	[illegible]	[illegible]	[illegible]	[illegible]	[illegible]	[illegible]	[illegible]	[illegible]	[illegible]	[illegible]	[illegible]	[illegible]	[illegible]	[illegible]	[illegible]	[illegible]	[illegible]	[illegible]	[illegible]	[illegible]	[illegible]	[illegible]	[illegible]	[illegible]	[illegible]	[illegible]
			Cervical	[illegible]	[illegible]	[illegible]	[illegible]	[illegible]	[illegible]	[illegible]	[illegible]	[illegible]	[illegible]	[illegible]	[illegible]	[illegible]	[illegible]	[illegible]	[illegible]	[illegible]	[illegible]	[illegible]	[illegible]	[illegible]	[illegible]	[illegible]	[illegible]	[illegible]	[illegible]	[illegible]	[illegible]	[illegible]	[illegible]	[illegible]	[illegible]	[illegible]	[illegible]	[illegible]	[illegible]
Fluoride application	Baseline	Maxilla	Edge	[illegible]	[illegible]	[illegible]	[illegible]	[illegible]	[illegible]	[illegible]	[illegible]	[illegible]	[illegible]	[illegible]	[illegible]	[illegible]	[illegible]	[illegible]	[illegible]	[illegible]	[illegible]	[illegible]	[illegible]	[illegible]	[illegible]	[illegible]	[illegible]	[illegible]	[illegible]	[illegible]	[illegible]	[illegible]	[illegible]	[illegible]	[illegible]	[illegible]	[illegible]	[illegible]	[illegible]
			Central	[illegible]	[illegible]	[illegible]	[illegible]	[illegible]	[illegible]	[illegible]	[illegible]	[illegible]	[illegible]	[illegible]	[illegible]	[illegible]	[illegible]	[illegible]	[illegible]	[illegible]	[illegible]	[illegible]	[illegible]	[illegible]	[illegible]	[illegible]	[illegible]	[illegible]	[illegible]	[illegible]	[illegible]	[illegible]	[illegible]	[illegible]	[illegible]	[illegible]	[illegible]	[illegible]	[illegible]
			Cervical	[illegible]	[illegible]	[illegible]	[illegible]	[illegible]	[illegible]	[illegible]	[illegible]	[illegible]	[illegible]	[illegible]	[illegible]	[illegible]	[illegible]	[illegible]	[illegible]	[illegible]	[illegible]	[illegible]	[illegible]	[illegible]	[illegible]	[illegible]	[illegible]	[illegible]	[illegible]	[illegible]	[illegible]	[illegible]	[illegible]	[illegible]	[illegible]	[illegible]	[illegible]	[illegible]	[illegible]
		Mandibular	Edge	[illegible]	[illegible]	[illegible]	[illegible]	[illegible]	[illegible]	[illegible]	[illegible]	[illegible]	[illegible]	[illegible]	[illegible]	[illegible]	[illegible]	[illegible]	[illegible]	[illegible]	[illegible]	[illegible]	[illegible]	[illegible]	[illegible]	[illegible]	[illegible]	[illegible]	[illegible]	[illegible]	[illegible]	[illegible]	[illegible]	[illegible]	[illegible]	[illegible]	[illegible]	[illegible]	[illegible]
			Central	[illegible]	[illegible]	[illegible]	[illegible]	[illegible]	[illegible]	[illegible]	[illegible]	[illegible]	[illegible]	[illegible]	[illegible]	[illegible]	[illegible]	[illegible]	[illegible]	[illegible]	[illegible]	[illegible]	[illegible]	[illegible]	[illegible]	[illegible]	[illegible]	[illegible]	[illegible]	[illegible]	[illegible]	[illegible]	[illegible]	[illegible]	[illegible]	[illegible]	[illegible]	[illegible]	[illegible]
			Cervical	[illegible]	[illegible]	[illegible]	[illegible]	[illegible]	[illegible]	[illegible]	[illegible]	[illegible]	[illegible]	[illegible]	[illegible]	[illegible]	[illegible]	[illegible]	[illegible]	[illegible]	[illegible]	[illegible]	[illegible]	[illegible]	[illegible]	[illegible]	[illegible]	[illegible]	[illegible]	[illegible]	[illegible]	[illegible]	[illegible]	[illegible]	[illegible]	[illegible]	[illegible]	[illegible]	[illegible]
	After 6 months	Maxilla	Edge	[illegible]	[illegible]	[illegible]	[illegible]	[illegible]	[illegible]	[illegible]	[illegible]	[illegible]	[illegible]	[illegible]	[illegible]	[illegible]	[illegible]	[illegible]	[illegible]	[illegible]	[illegible]	[illegible]	[illegible]	[illegible]	[illegible]	[illegible]	[illegible]	[illegible]	[illegible]	[illegible]	[illegible]	[illegible]	[illegible]	[illegible]	[illegible]	[illegible]	[illegible]	[illegible]	[illegible]
			Central	[illegible]	[illegible]	[illegible]	[illegible]	[illegible]	[illegible]	[illegible]	[illegible]	[illegible]	[illegible]	[illegible]	[illegible]	[illegible]	[illegible]	[illegible]	[illegible]	[illegible]	[illegible]	[illegible]	[illegible]	[illegible]	[illegible]	[illegible]	[illegible]	[illegible]	[illegible]	[illegible]	[illegible]	[illegible]	[illegible]	[illegible]	[illegible]	[illegible]	[illegible]	[illegible]	[illegible]
			Cervical	[illegible]	[illegible]	[illegible]	[illegible]	[illegible]	[illegible]	[illegible]	[illegible]	[illegible]	[illegible]	[illegible]	[illegible]	[illegible]	[illegible]	[illegible]	[illegible]	[illegible]	[illegible]	[illegible]	[illegible]	[illegible]	[illegible]	[illegible]	[illegible]	[illegible]	[illegible]	[illegible]	[illegible]	[illegible]	[illegible]	[illegible]	[illegible]	[illegible]	[illegible]	[illegible]	[illegible]
		Mandibular	Edge	[illegible]	[illegible]	[illegible]	[illegible]	[illegible]	[illegible]	[illegible]	[illegible]	[illegible]	[illegible]	[illegible]	[illegible]	[illegible]	[illegible]	[illegible]	[illegible]	[illegible]	[illegible]	[illegible]	[illegible]	[illegible]	[illegible]	[illegible]	[illegible]	[illegible]	[illegible]	[illegible]	[illegible]	[illegible]	[illegible]	[illegible]	[illegible]	[illegible]	[illegible]	[illegible]	[illegible]
			Central	[illegible]	[illegible]	[illegible]	[illegible]	[illegible]	[illegible]	[illegible]	[illegible]	[illegible]	[illegible]	[illegible]	[illegible]	[illegible]	[illegible]	[illegible]	[illegible]	[illegible]	[illegible]	[illegible]	[illegible]	[illegible]	[illegible]	[illegible]	[illegible]	[illegible]	[illegible]	[illegible]	[illegible]	[illegible]	[illegible]	[illegible]	[illegible]	[illegible]	[illegible]	[illegible]	[illegible]
			Cervical	[illegible]	[illegible]	[illegible]	[illegible]	[illegible]	[illegible]	[illegible]	[illegible]	[illegible]	[illegible]	[illegible]	[illegible]	[illegible]	[illegible]	[illegible]	[illegible]	[illegible]	[illegible]	[illegible]	[illegible]	[illegible]	[illegible]	[illegible]	[illegible]	[illegible]	[illegible]	[illegible]	[illegible]	[illegible]	[illegible]	[illegible]	[illegible]	[illegible]	[illegible]	[illegible]	[illegible]

				ΔFmax(%)																																			
				Right																		Left																	
				3						2						1						1						2						3					
				Distal		Central		Medial		Distal		Central		Medial		Distal		Central		Medial		Medial		Central		Distal		Medial		Central		Distal		Medial		Central		Distal	
				Mean	Median	Mean	Median	Mean	Median	Mean	Median	Mean	Median	Mean	Median	Mean	Median	Mean	Median	Mean	Median	Mean	Median	Mean	Median	Mean	Median	Mean	Median	Mean	Median	Mean	Median	Mean	Median	Mean	Median	Mean	Median
Tooth surface disinfection	Baseline	Maxilla	Edge	[illegible]	[illegible]	[illegible]	[illegible]	[illegible]	[illegible]	[illegible]	[illegible]	[illegible]	[illegible]	[illegible]	[illegible]	[illegible]	[illegible]	[illegible]	[illegible]	[illegible]	[illegible]	[illegible]	[illegible]	[illegible]	[illegible]	[illegible]	[illegible]	[illegible]	[illegible]	[illegible]	[illegible]	[illegible]	[illegible]	[illegible]	[illegible]	[illegible]	[illegible]	[illegible]	[illegible]
			Central	[illegible]	[illegible]	[illegible]	[illegible]	[illegible]	[illegible]	[illegible]	[illegible]	[illegible]	[illegible]	[illegible]	[illegible]	[illegible]	[illegible]	[illegible]	[illegible]	[illegible]	[illegible]	[illegible]	[illegible]	[illegible]	[illegible]	[illegible]	[illegible]	[illegible]	[illegible]	[illegible]	[illegible]	[illegible]	[illegible]	[illegible]	[illegible]	[illegible]	[illegible]	[illegible]	[illegible]
			Cervical	[illegible]	[illegible]	[illegible]	[illegible]	[illegible]	[illegible]	[illegible]	[illegible]	[illegible]	[illegible]	[illegible]	[illegible]	[illegible]	[illegible]	[illegible]	[illegible]	[illegible]	[illegible]	[illegible]	[illegible]	[illegible]	[illegible]	[illegible]	[illegible]	[illegible]	[illegible]	[illegible]	[illegible]	[illegible]	[illegible]	[illegible]	[illegible]	[illegible]	[illegible]	[illegible]	[illegible]
		Mandibular	Edge	[illegible]	[illegible]	[illegible]	[illegible]	[illegible]	[illegible]	[illegible]	[illegible]	[illegible]	[illegible]	[illegible]	[illegible]	[illegible]	[illegible]	[illegible]	[illegible]	[illegible]	[illegible]	[illegible]	[illegible]	[illegible]	[illegible]	[illegible]	[illegible]	[illegible]	[illegible]	[illegible]	[illegible]	[illegible]	[illegible]	[illegible]	[illegible]	[illegible]	[illegible]	[illegible]	[illegible]
			Central	[illegible]	[illegible]	[illegible]	[illegible]	[illegible]	[illegible]	[illegible]	[illegible]	[illegible]	[illegible]	[illegible]	[illegible]	[illegible]	[illegible]	[illegible]	[illegible]	[illegible]	[illegible]	[illegible]	[illegible]	[illegible]	[illegible]	[illegible]	[illegible]	[illegible]	[illegible]	[illegible]	[illegible]	[illegible]	[illegible]	[illegible]	[illegible]	[illegible]	[illegible]	[illegible]	[illegible]
			Cervical	[illegible]	[illegible]	[illegible]	[illegible]	[illegible]	[illegible]	[illegible]	[illegible]	[illegible]	[illegible]	[illegible]	[illegible]	[illegible]	[illegible]	[illegible]	[illegible]	[illegible]	[illegible]	[illegible]	[illegible]	[illegible]	[illegible]	[illegible]	[illegible]	[illegible]	[illegible]	[illegible]	[illegible]	[illegible]	[illegible]	[illegible]	[illegible]	[illegible]	[illegible]	[illegible]	[illegible]
	After 6 months	Maxilla	Edge	[illegible]	[illegible]	[illegible]	[illegible]	[illegible]	[illegible]	[illegible]	[illegible]	[illegible]	[illegible]	[illegible]	[illegible]	[illegible]	[illegible]	[illegible]	[illegible]	[illegible]	[illegible]	[illegible]	[illegible]	[illegible]	[illegible]	[illegible]	[illegible]	[illegible]	[illegible]	[illegible]	[illegible]	[illegible]	[illegible]	[illegible]	[illegible]	[illegible]	[illegible]	[illegible]	[illegible]
			Central	[illegible]	[illegible]	[illegible]	[illegible]	[illegible]	[illegible]	[illegible]	[illegible]	[illegible]	[illegible]	[illegible]	[illegible]	[illegible]	[illegible]	[illegible]	[illegible]	[illegible]	[illegible]	[illegible]	[illegible]	[illegible]	[illegible]	[illegible]	[illegible]	[illegible]	[illegible]	[illegible]	[illegible]	[illegible]	[illegible]	[illegible]	[illegible]	[illegible]	[illegible]	[illegible]	[illegible]
			Cervical	[illegible]	[illegible]	[illegible]	[illegible]	[illegible]	[illegible]	[illegible]	[illegible]	[illegible]	[illegible]	[illegible]	[illegible]	[illegible]	[illegible]	[illegible]	[illegible]	[illegible]	[illegible]	[illegible]	[illegible]	[illegible]	[illegible]	[illegible]	[illegible]	[illegible]	[illegible]	[illegible]	[illegible]	[illegible]	[illegible]	[illegible]	[illegible]	[illegible]	[illegible]	[illegible]	[illegible]
		Mandibular	Edge	[illegible]	[illegible]	[illegible]	[illegible]	[illegible]	[illegible]	[illegible]	[illegible]	[illegible]	[illegible]	[illegible]	[illegible]	[illegible]	[illegible]	[illegible]	[illegible]	[illegible]	[illegible]	[illegible]	[illegible]	[illegible]	[illegible]	[illegible]	[illegible]	[illegible]	[illegible]	[illegible]	[illegible]	[illegible]	[illegible]	[illegible]	[illegible]	[illegible]	[illegible]	[illegible]	[illegible]
			Central	[illegible]	[illegible]	[illegible]	[illegible]	[illegible]	[illegible]	[illegible]	[illegible]	[illegible]	[illegible]	[illegible]	[illegible]	[illegible]	[illegible]	[illegible]	[illegible]	[illegible]	[illegible]	[illegible]	[illegible]	[illegible]	[illegible]	[illegible]	[illegible]	[illegible]	[illegible]	[illegible]	[illegible]	[illegible]	[illegible]	[illegible]	[illegible]	[illegible]	[illegible]	[illegible]	[illegible]
			Cervical	[illegible]	[illegible]	[illegible]	[illegible]	[illegible]	[illegible]	[illegible]	[illegible]	[illegible]	[illegible]	[illegible]	[illegible]	[illegible]	[illegible]	[illegible]	[illegible]	[illegible]	[illegible]	[illegible]	[illegible]	[illegible]	[illegible]	[illegible]	[illegible]	[illegible]	[illegible]	[illegible]	[illegible]	[illegible]	[illegible]	[illegible]	[illegible]	[illegible]	[illegible]	[illegible]	[illegible]
Fluoride application	Baseline	Maxilla	Edge	[illegible]	[illegible]	[illegible]	[illegible]	[illegible]	[illegible]	[illegible]	[illegible]	[illegible]	[illegible]	[illegible]	[illegible]	[illegible]	[illegible]	[illegible]	[illegible]	[illegible]	[illegible]	[illegible]	[illegible]	[illegible]	[illegible]	[illegible]	[illegible]	[illegible]	[illegible]	[illegible]	[illegible]	[illegible]	[illegible]	[illegible]	[illegible]	[illegible]	[illegible]	[illegible]	[illegible]
			Central	[illegible]	[illegible]	[illegible]	[illegible]	[illegible]	[illegible]	[illegible]	[illegible]	[illegible]	[illegible]	[illegible]	[illegible]	[illegible]	[illegible]	[illegible]	[illegible]	[illegible]	[illegible]	[illegible]	[illegible]	[illegible]	[illegible]	[illegible]	[illegible]	[illegible]	[illegible]	[illegible]	[illegible]	[illegible]	[illegible]	[illegible]	[illegible]	[illegible]	[illegible]	[illegible]	[illegible]
			Cervical	[illegible]	[illegible]	[illegible]	[illegible]	[illegible]	[illegible]	[illegible]	[illegible]	[illegible]	[illegible]	[illegible]	[illegible]	[illegible]	[illegible]	[illegible]	[illegible]	[illegible]	[illegible]	[illegible]	[illegible]	[illegible]	[illegible]	[illegible]	[illegible]	[illegible]	[illegible]	[illegible]	[illegible]	[illegible]	[illegible]	[illegible]	[illegible]	[illegible]	[illegible]	[illegible]	[illegible]
		Mandibular	Edge	[illegible]	[illegible]	[illegible]	[illegible]	[illegible]	[illegible]	[illegible]	[illegible]	[illegible]	[illegible]	[illegible]	[illegible]	[illegible]	[illegible]	[illegible]	[illegible]	[illegible]	[illegible]	[illegible]	[illegible]	[illegible]	[illegible]	[illegible]	[illegible]	[illegible]	[illegible]	[illegible]	[illegible]	[illegible]	[illegible]	[illegible]	[illegible]	[illegible]	[illegible]	[illegible]	[illegible]
			Central	[illegible]	[illegible]	[illegible]	[illegible]	[illegible]	[illegible]	[illegible]	[illegible]	[illegible]	[illegible]	[illegible]	[illegible]	[illegible]	[illegible]	[illegible]	[illegible]	[illegible]	[illegible]	[illegible]	[illegible]	[illegible]	[illegible]	[illegible]	[illegible]	[illegible]	[illegible]	[illegible]	[illegible]	[illegible]	[illegible]	[illegible]	[illegible]	[illegible]	[illegible]	[illegible]	[illegible]
			Cervical	[illegible]	[illegible]	[illegible]	[illegible]	[illegible]	[illegible]	[illegible]	[illegible]	[illegible]	[illegible]	[illegible]	[illegible]	[illegible]	[illegible]	[illegible]	[illegible]	[illegible]	[illegible]	[illegible]	[illegible]	[illegible]	[illegible]	[illegible]	[illegible]	[illegible]	[illegible]	[illegible]	[illegible]	[illegible]	[illegible]	[illegible]	[illegible]	[illegible]	[illegible]	[illegible]	[illegible]
	After 6 months	Maxilla	Edge	[illegible]	[illegible]	[illegible]	[illegible]	[illegible]	[illegible]	[illegible]	[illegible]	[illegible]	[illegible]	[illegible]	[illegible]	[illegible]	[illegible]	[illegible]	[illegible]	[illegible]	[illegible]	[illegible]	[illegible]	[illegible]	[illegible]	[illegible]	[illegible]	[illegible]	[illegible]	[illegible]	[illegible]	[illegible]	[illegible]	[illegible]	[illegible]	[illegible]	[illegible]	[illegible]	[illegible]
			Central	[illegible]	[illegible]	[illegible]	[illegible]	[illegible]	[illegible]	[illegible]	[illegible]	[illegible]	[illegible]	[illegible]	[illegible]	[illegible]	[illegible]	[illegible]	[illegible]	[illegible]	[illegible]	[illegible]	[illegible]	[illegible]	[illegible]	[illegible]	[illegible]	[illegible]	[illegible]	[illegible]	[illegible]	[illegible]	[illegible]	[illegible]	[illegible]	[illegible]	[illegible]	[illegible]	[illegible]
			Cervical	[illegible]	[illegible]	[illegible]	[illegible]	[illegible]	[illegible]	[illegible]	[illegible]	[illegible]	[illegible]	[illegible]	[illegible]	[illegible]	[illegible]	[illegible]	[illegible]	[illegible]	[illegible]	[illegible]	[illegible]	[illegible]	[illegible]	[illegible]	[illegible]	[illegible]	[illegible]	[illegible]	[illegible]	[illegible]	[illegible]	[illegible]	[illegible]	[illegible]	[illegible]	[illegible]	[illegible]
		Mandibular	Edge	[illegible]	[illegible]	[illegible]	[illegible]	[illegible]	[illegible]	[illegible]	[illegible]	[illegible]	[illegible]	[illegible]	[illegible]	[illegible]	[illegible]	[illegible]	[illegible]	[illegible]	[illegible]	[illegible]	[illegible]	[illegible]	[illegible]	[illegible]	[illegible]	[illegible]	[illegible]	[illegible]	[illegible]	[illegible]	[illegible]	[illegible]	[illegible]	[illegible]	[illegible]	[illegible]	[illegible]
			Central	[illegible]	[illegible]	[illegible]	[illegible]	[illegible]	[illegible]	[illegible]	[illegible]	[illegible]	[illegible]	[illegible]	[illegible]	[illegible]	[illegible]	[illegible]	[illegible]	[illegible]	[illegible]	[illegible]	[illegible]	[illegible]	[illegible]	[illegible]	[illegible]	[illegible]	[illegible]	[illegible]	[illegible]	[illegible]	[illegible]	[illegible]	[illegible]	[illegible]	[illegible]	[illegible]	[illegible]
			Cervical	[illegible]	[illegible]	[illegible]	[illegible]	[illegible]	[illegible]	[illegible]	[illegible]	[illegible]	[illegible]	[illegible]	[illegible]	[illegible]	[illegible]	[illegible]	[illegible]	[illegible]	[illegible]	[illegible]	[illegible]	[illegible]	[illegible]	[illegible]	[illegible]	[illegible]	[illegible]	[illegible]	[illegible]	[illegible]	[illegible]	[illegible]	[illegible]	[illegible]	[illegible]	[illegible]	[illegible]

				ΔQ(%×mm^2)																																			
				Right																		Left																	
				3						2						1						1						2						3					
				Distal		Central		Medial		Distal		Central		Medial		Distal		Central		Medial		Medial		Central		Distal		Medial		Central		Distal		Medial		Central		Distal	
				Mean	Median	Mean	Median	Mean	Median	Mean	Median	Mean	Median	Mean	Median	Mean	Median	Mean	Median	Mean	Median	Mean	Median	Mean	Median	Mean	Median	Mean	Median	Mean	Median	Mean	Median	Mean	Median	Mean	Median	Mean	Median
Tooth surface disinfection	Baseline	Maxilla	Edge	[illegible]	[illegible]	[illegible]	[illegible]	[illegible]	[illegible]	[illegible]	[illegible]	[illegible]	[illegible]	[illegible]	[illegible]	[illegible]	[illegible]	[illegible]	[illegible]	[illegible]	[illegible]	[illegible]	[illegible]	[illegible]	[illegible]	[illegible]	[illegible]	[illegible]	[illegible]	[illegible]	[illegible]	[illegible]	[illegible]	[illegible]	[illegible]	[illegible]	[illegible]	[illegible]	[illegible]
			Central	[illegible]	[illegible]	[illegible]	[illegible]	[illegible]	[illegible]	[illegible]	[illegible]	[illegible]	[illegible]	[illegible]	[illegible]	[illegible]	[illegible]	[illegible]	[illegible]	[illegible]	[illegible]	[illegible]	[illegible]	[illegible]	[illegible]	[illegible]	[illegible]	[illegible]	[illegible]	[illegible]	[illegible]	[illegible]	[illegible]	[illegible]	[illegible]	[illegible]	[illegible]	[illegible]	[illegible]
			Cervical	[illegible]	[illegible]	[illegible]	[illegible]	[illegible]	[illegible]	[illegible]	[illegible]	[illegible]	[illegible]	[illegible]	[illegible]	[illegible]	[illegible]	[illegible]	[illegible]	[illegible]	[illegible]	[illegible]	[illegible]	[illegible]	[illegible]	[illegible]	[illegible]	[illegible]	[illegible]	[illegible]	[illegible]	[illegible]	[illegible]	[illegible]	[illegible]	[illegible]	[illegible]	[illegible]	[illegible]
		Mandibular	Edge	[illegible]	[illegible]	[illegible]	[illegible]	[illegible]	[illegible]	[illegible]	[illegible]	[illegible]	[illegible]	[illegible]	[illegible]	[illegible]	[illegible]	[illegible]	[illegible]	[illegible]	[illegible]	[illegible]	[illegible]	[illegible]	[illegible]	[illegible]	[illegible]	[illegible]	[illegible]	[illegible]	[illegible]	[illegible]	[illegible]	[illegible]	[illegible]	[illegible]	[illegible]	[illegible]	[illegible]
			Central	[illegible]	[illegible]	[illegible]	[illegible]	[illegible]	[illegible]	[illegible]	[illegible]	[illegible]	[illegible]	[illegible]	[illegible]	[illegible]	[illegible]	[illegible]	[illegible]	[illegible]	[illegible]	[illegible]	[illegible]	[illegible]	[illegible]	[illegible]	[illegible]	[illegible]	[illegible]	[illegible]	[illegible]	[illegible]	[illegible]	[illegible]	[illegible]	[illegible]	[illegible]	[illegible]	[illegible]
			Cervical	[illegible]	[illegible]	[illegible]	[illegible]	[illegible]	[illegible]	[illegible]	[illegible]	[illegible]	[illegible]	[illegible]	[illegible]	[illegible]	[illegible]	[illegible]	[illegible]	[illegible]	[illegible]	[illegible]	[illegible]	[illegible]	[illegible]	[illegible]	[illegible]	[illegible]	[illegible]	[illegible]	[illegible]	[illegible]	[illegible]	[illegible]	[illegible]	[illegible]	[illegible]	[illegible]	[illegible]
	After treatment	Maxilla	Edge	[illegible]	[illegible]	[illegible]	[illegible]	[illegible]	[illegible]	[illegible]	[illegible]	[illegible]	[illegible]	[illegible]	[illegible]	[illegible]	[illegible]	[illegible]	[illegible]	[illegible]	[illegible]	[illegible]	[illegible]	[illegible]	[illegible]	[illegible]	[illegible]	[illegible]	[illegible]	[illegible]	[illegible]	[illegible]	[illegible]	[illegible]	[illegible]	[illegible]	[illegible]	[illegible]	[illegible]
			Central	[illegible]	[illegible]	[illegible]	[illegible]	[illegible]	[illegible]	[illegible]	[illegible]	[illegible]	[illegible]	[illegible]	[illegible]	[illegible]	[illegible]	[illegible]	[illegible]	[illegible]	[illegible]	[illegible]	[illegible]	[illegible]	[illegible]	[illegible]	[illegible]	[illegible]	[illegible]	[illegible]	[illegible]	[illegible]	[illegible]	[illegible]	[illegible]	[illegible]	[illegible]	[illegible]	[illegible]
			Cervical	[illegible]	[illegible]	[illegible]	[illegible]	[illegible]	[illegible]	[illegible]	[illegible]	[illegible]	[illegible]	[illegible]	[illegible]	[illegible]	[illegible]	[illegible]	[illegible]	[illegible]	[illegible]	[illegible]	[illegible]	[illegible]	[illegible]	[illegible]	[illegible]	[illegible]	[illegible]	[illegible]	[illegible]	[illegible]	[illegible]	[illegible]	[illegible]	[illegible]	[illegible]	[illegible]	[illegible]
		Mandibular	Edge	[illegible]	[illegible]	[illegible]	[illegible]	[illegible]	[illegible]	[illegible]	[illegible]	[illegible]	[illegible]	[illegible]	[illegible]	[illegible]	[illegible]	[illegible]	[illegible]	[illegible]	[illegible]	[illegible]	[illegible]	[illegible]	[illegible]	[illegible]	[illegible]	[illegible]	[illegible]	[illegible]	[illegible]	[illegible]	[illegible]	[illegible]	[illegible]	[illegible]	[illegible]	[illegible]	[illegible]
			Central	[illegible]	[illegible]	[illegible]	[illegible]	[illegible]	[illegible]	[illegible]	[illegible]	[illegible]	[illegible]	[illegible]	[illegible]	[illegible]	[illegible]	[illegible]	[illegible]	[illegible]	[illegible]	[illegible]	[illegible]	[illegible]	[illegible]	[illegible]	[illegible]	[illegible]	[illegible]	[illegible]	[illegible]	[illegible]	[illegible]	[illegible]	[illegible]	[illegible]	[illegible]	[illegible]	[illegible]
			Cervical	[illegible]	[illegible]	[illegible]	[illegible]	[illegible]	[illegible]	[illegible]	[illegible]	[illegible]	[illegible]	[illegible]	[illegible]	[illegible]	[illegible]	[illegible]	[illegible]	[illegible]	[illegible]	[illegible]	[illegible]	[illegible]	[illegible]	[illegible]	[illegible]	[illegible]	[illegible]	[illegible]	[illegible]	[illegible]	[illegible]	[illegible]	[illegible]	[illegible]	[illegible]	[illegible]	[illegible]
Fluoride application	Baseline	Maxilla	Edge	[illegible]	[illegible]	[illegible]	[illegible]	[illegible]	[illegible]	[illegible]	[illegible]	[illegible]	[illegible]	[illegible]	[illegible]	[illegible]	[illegible]	[illegible]	[illegible]	[illegible]	[illegible]	[illegible]	[illegible]	[illegible]	[illegible]	[illegible]	[illegible]	[illegible]	[illegible]	[illegible]	[illegible]	[illegible]	[illegible]	[illegible]	[illegible]	[illegible]	[illegible]	[illegible]	[illegible]
			Central	[illegible]	[illegible]	[illegible]	[illegible]	[illegible]	[illegible]	[illegible]	[illegible]	[illegible]	[illegible]	[illegible]	[illegible]	[illegible]	[illegible]	[illegible]	[illegible]	[illegible]	[illegible]	[illegible]	[illegible]	[illegible]	[illegible]	[illegible]	[illegible]	[illegible]	[illegible]	[illegible]	[illegible]	[illegible]	[illegible]	[illegible]	[illegible]	[illegible]	[illegible]	[illegible]	[illegible]
			Cervical	[illegible]	[illegible]	[illegible]	[illegible]	[illegible]	[illegible]	[illegible]	[illegible]	[illegible]	[illegible]	[illegible]	[illegible]	[illegible]	[illegible]	[illegible]	[illegible]	[illegible]	[illegible]	[illegible]	[illegible]	[illegible]	[illegible]	[illegible]	[illegible]	[illegible]	[illegible]	[illegible]	[illegible]	[illegible]	[illegible]	[illegible]	[illegible]	[illegible]	[illegible]	[illegible]	[illegible]
		Mandibular	Edge	[illegible]	[illegible]	[illegible]	[illegible]	[illegible]	[illegible]	[illegible]	[illegible]	[illegible]	[illegible]	[illegible]	[illegible]	[illegible]	[illegible]	[illegible]	[illegible]	[illegible]	[illegible]	[illegible]	[illegible]	[illegible]	[illegible]	[illegible]	[illegible]	[illegible]	[illegible]	[illegible]	[illegible]	[illegible]	[illegible]	[illegible]	[illegible]	[illegible]	[illegible]	[illegible]	[illegible]
			Central	[illegible]	[illegible]	[illegible]	[illegible]	[illegible]	[illegible]	[illegible]	[illegible]	[illegible]	[illegible]	[illegible]	[illegible]	[illegible]	[illegible]	[illegible]	[illegible]	[illegible]	[illegible]	[illegible]	[illegible]	[illegible]	[illegible]	[illegible]	[illegible]	[illegible]	[illegible]	[illegible]	[illegible]	[illegible]	[illegible]	[illegible]	[illegible]	[illegible]	[illegible]	[illegible]	[illegible]
			Cervical	[illegible]	[illegible]	[illegible]	[illegible]	[illegible]	[illegible]	[illegible]	[illegible]	[illegible]	[illegible]	[illegible]	[illegible]	[illegible]	[illegible]	[illegible]	[illegible]	[illegible]	[illegible]	[illegible]	[illegible]	[illegible]	[illegible]	[illegible]	[illegible]	[illegible]	[illegible]	[illegible]	[illegible]	[illegible]	[illegible]	[illegible]	[illegible]	[illegible]	[illegible]	[illegible]	[illegible]
	After treatment	Maxilla	Edge	[illegible]	[illegible]	[illegible]	[illegible]	[illegible]	[illegible]	[illegible]	[illegible]	[illegible]	[illegible]	[illegible]	[illegible]	[illegible]	[illegible]	[illegible]	[illegible]	[illegible]	[illegible]	[illegible]	[illegible]	[illegible]	[illegible]	[illegible]	[illegible]	[illegible]	[illegible]	[illegible]	[illegible]	[illegible]	[illegible]	[illegible]	[illegible]	[illegible]	[illegible]	[illegible]	[illegible]
			Central	[illegible]	[illegible]	[illegible]	[illegible]	[illegible]	[illegible]	[illegible]	[illegible]	[illegible]	[illegible]	[illegible]	[illegible]	[illegible]	[illegible]	[illegible]	[illegible]	[illegible]	[illegible]	[illegible]	[illegible]	[illegible]	[illegible]	[illegible]	[illegible]	[illegible]	[illegible]	[illegible]	[illegible]	[illegible]	[illegible]	[illegible]	[illegible]	[illegible]	[illegible]	[illegible]	[illegible]
			Cervical	[illegible]	[illegible]	[illegible]	[illegible]	[illegible]	[illegible]	[illegible]	[illegible]	[illegible]	[illegible]	[illegible]	[illegible]	[illegible]	[illegible]	[illegible]	[illegible]	[illegible]	[illegible]	[illegible]	[illegible]	[illegible]	[illegible]	[illegible]	[illegible]	[illegible]	[illegible]	[illegible]	[illegible]	[illegible]	[illegible]	[illegible]	[illegible]	[illegible]	[illegible]	[illegible]	[illegible]
		Mandibular	Edge	[illegible]	[illegible]	[illegible]	[illegible]	[illegible]	[illegible]	[illegible]	[illegible]	[illegible]	[illegible]	[illegible]	[illegible]	[illegible]	[illegible]	[illegible]	[illegible]	[illegible]	[illegible]	[illegible]	[illegible]	[illegible]	[illegible]	[illegible]	[illegible]	[illegible]	[illegible]	[illegible]	[illegible]	[illegible]	[illegible]	[illegible]	[illegible]	[illegible]	[illegible]	[illegible]	[illegible]
			Central	[illegible]	[illegible]	[illegible]	[illegible]	[illegible]	[illegible]	[illegible]	[illegible]	[illegible]	[illegible]	[illegible]	[illegible]	[illegible]	[illegible]	[illegible]	[illegible]	[illegible]	[illegible]	[illegible]	[illegible]	[illegible]	[illegible]	[illegible]	[illegible]	[illegible]	[illegible]	[illegible]	[illegible]	[illegible]	[illegible]	[illegible]	[illegible]	[illegible]	[illegible]	[illegible]	[illegible]
			Cervical	[illegible]	[illegible]	[illegible]	[illegible]	[illegible]	[illegible]	[illegible]	[illegible]	[illegible]	[illegible]	[illegible]	[illegible]	[illegible]	[illegible]	[illegible]	[illegible]	[illegible]	[illegible]	[illegible]	[illegible]	[illegible]	[illegible]	[illegible]	[illegible]	[illegible]	[illegible]	[illegible]	[illegible]	[illegible]	[illegible]	[illegible]	[illegible]	[illegible]	[illegible]	[illegible]	[illegible]

Table S4: Results of changes of QLF parameters by mixed effect modeling with risk factors supposed to be dental caries.

			White spot area(mm^2)		ΔF(%)		ΔFmax(%)		ΔQ(%×mm^2)	
			Coefficient (95% CI)	P-value	Coefficient (95% CI)	P-value	Coefficient (95% CI)	P-value	Coefficient (95% CI)	P-value
Fixed effedct										
Intercept			0.231(0.169-0.294)	0	2.133(2.091-2.174)	0	2.535(2.474-2.596)	0	1.907(1.188-2.626)	0
Treatment	Tooth surface disinfection		-0.109(-0.17--0.048)	0	-0.058(-0.106--0.01)	0.018	-0.187(-0.251--0.122)	0	-1.089(-1.8--0.377)	0.003
	Fluoride application		Reference		Reference		Reference		Reference	
Time	After 6 months		-0.015(-0.033-0.004)	0.124	-0.025(-0.036--0.014)	0	-0.079(-0.1--0.057)	0	-0.111(-0.32-0.098)	0.297
	Baseine		Reference		Reference		Reference		Reference	
Tooth type	Maxillary	1	0.039(-0.017-0.096)	0.169	-0.099(-0.125--0.073)	0	-0.125(-0.164--0.087)	0	0.206(-0.434-0.847)	0.527
		2	0.008(-0.05-0.067)	0.777	-0.042(-0.069--0.015)	0.002	-0.063(-0.103--0.022)	0.002	0.242(-0.421-0.905)	0.474
		3	0.061(0.009-0.113)	0.022	-0.022(-0.046-0.002)	0.072	-0.014(-0.05-0.022)	0.434	0.68(0.087-1.274)	0.025
	Mandibular	1	-0.056(-0.11--0.003)	0.039	-0.045(-0.07--0.021)	0	-0.072(-0.109--0.035)	0	-0.493(-1.1-0.114)	0.111
		2	-0.052(-0.108-0.004)	0.068	-0.039(-0.065--0.013)	0.003	-0.051(-0.09--0.012)	0.01	-0.454(-1.089-0.181)	0.161
		3	Reference		Reference		Reference		Reference	
Biofilm area of the entire tooth surface(mm^2)			0.011(0.009-0.014)	0	0.005(0.003-0.006)	0	0.009(0.007-0.011)	0	0.128(0.103-0.152)	0
Random effect										
Level1(subhject)			0.004(0.002-0.009)	0.02	0.003(0.001-0.006)	0.004	0.005(0.003-0.01)	0.005	0.527(0.23-1.203)	0.018
Level2(Tooth)			0.006(0.004-0.01)	0	0.001(0.001-0.002)	0.001	0.001(0.00007245-0.006)	0.383	0.808(0.501-1.303)	0
Level3 (ordination)			0.043(0.037-0.049)	0	0.009(0.008-0.011)	0	0.023(0.018-0.029)	0	5.237(4.544-6.036)	0
Fitness index										
AICC			2872.026		-2102.342		2794.049		22335.927	
BIC			2903.504		-2070.872		2825.52		22367.405	
Distribution			Normal, Link :Identity		Γ, Link :log		Γ, Link :log		Γ, Link :log	

Table S5. The summary statistics of the alpha diversity indices

Treatment	Time	Sample No	ID	observed	chao1	diversity_inverse_simpson	diversity_gini_simpson	diversity_shannon	diversity_fisher	diversity_coverage	evenness_camargo	evenness_pielou	evenness_simpson	evenness_evar	evenness_bulla	dominance_dbp	dominance_dmn	dominance_absolute	dominance_relative	dominance_simpson	dominance_core_abundance	dominance_gini	rarity_log_modulo_skewness	rarity_low_abundance	rarity_rare_abundance
Tooth surface disinfection	Baseline	Sample 1	100401	219	237.75	11.83	0.92	3.18	29.34	4	0.483	0.589	0.054	0.150	0.236	0.164	0.299	8373	0.164	0.085	0.787	0.957	2.060	0.072	0.113
		Sample 2	101301	226	239.80	22.04	0.95	3.66	29.98	8	0.591	0.674	0.098	0.130	0.267	0.103	0.188	5801	0.103	0.045	0.768	0.943	2.057	0.071	0.091
		Sample 3	102001	213	219.67	15.02	0.93	3.39	28.00	6	0.668	0.632	0.071	0.143	0.254	0.164	0.272	9235	0.164	0.067	0.831	0.952	2.059	0.070	0.071
		Sample 4	102701	197	209.04	5.96	0.83	2.43	25.85	2	0.648	0.461	0.030	0.155	0.157	0.261	0.517	13744	0.261	0.168	0.888	0.978	2.061	0.048	0.025
		Sample 5	100301	254	262.50	6.24	0.84	3.15	34.86	3	0.663	0.568	0.025	0.184	0.273	0.380	0.459	19316	0.380	0.160	0.786	0.941	2.059	0.097	0.103
		Sample 6	101001	315	321.77	11.34	0.91	3.36	42.38	4	0.263	0.584	0.036	0.165	0.231	0.221	0.358	15850	0.221	0.088	0.589	0.941	2.061	0.104	0.316
		Sample 7	100501	123	129.00	11.84	0.92	2.94	16.14	4	0.742	0.610	0.096	0.127	0.224	0.153	0.300	5043	0.153	0.084	0.879	0.973	2.056	0.035	0.020
		Sample 8	102101	159	166.58	14.71	0.93	3.28	21.25	5	0.720	0.647	0.093	0.138	0.269	0.144	0.270	5450	0.144	0.068	0.847	0.960	2.058	0.047	0.060
		Sample 9	102501	180	193.33	18.70	0.95	3.66	24.48	7	0.566	0.706	0.104	0.148	0.337	0.155	0.258	5923	0.155	0.053	0.749	0.939	2.057	0.061	0.127
		Sample 10	100601	213	216.67	9.42	0.89	3.23	29.72	4	0.652	0.603	0.044	0.184	0.270	0.276	0.394	10620	0.276	0.106	0.856	0.950	2.061	0.080	0.075
		Sample 11	101101	180	187.65	11.77	0.92	3.16	24.30	4	0.572	0.608	0.065	0.145	0.249	0.175	0.344	7007	0.175	0.085	0.854	0.961	2.060	0.057	0.041
	After 6 Months	Sample 12	100602	214	226.65	17.03	0.94	3.50	27.92	6	0.623	0.652	0.080	0.129	0.280	0.141	0.230	8373	0.141	0.059	0.751	0.947	2.059	0.065	0.085
		Sample 13	100502	150	185.00	5.42	0.82	2.46	21.91	2	0.872	0.492	0.036	0.158	0.185	0.376	0.536	7731	0.376	0.185	0.913	0.978	2.060	0.039	0.043
		Sample 14	101302	204	218.88	5.40	0.81	2.65	26.35	2	0.788	0.499	0.026	0.152	0.187	0.398	0.500	24152	0.398	0.185	0.894	0.970	2.060	0.066	0.040
		Sample 15	100302	197	200.79	13.70	0.93	3.31	26.37	5	0.583	0.626	0.070	0.143	0.254	0.167	0.297	7740	0.167	0.073	0.850	0.957	2.056	0.056	0.040
		Sample 16	101102	226	236.62	4.14	0.76	2.45	29.75	2	0.788	0.452	0.018	0.160	0.173	0.467	0.577	27650	0.467	0.241	0.907	0.971	2.060	0.065	0.057
		Sample 17	100402	188	195.43	13.17	0.92	3.18	24.20	5	0.675	0.607	0.070	0.125	0.223	0.185	0.306	10592	0.185	0.076	0.849	0.963	2.060	0.050	0.077
		Sample 18	101002	215	224.50	11.86	0.92	3.22	29.50	5	0.622	0.599	0.055	0.161	0.222	0.208	0.337	8967	0.208	0.084	0.815	0.957	2.060	0.081	0.085
		Sample 19	102102	189	198.55	11.47	0.91	3.19	26.05	5	0.651	0.608	0.061	0.146	0.239	0.231	0.332	8517	0.231	0.087	0.881	0.961	2.060	0.059	0.044
		Sample 20	102002	269	277.50	18.70	0.95	3.74	38.66	7	0.482	0.668	0.070	0.176	0.296	0.138	0.246	5605	0.138	0.053	0.763	0.928	2.060	0.091	0.093
		Sample 21	102702	230	241.18	18.00	0.94	3.55	33.37	7	0.594	0.652	0.078	0.176	0.268	0.110	0.219	3611	0.110	0.056	0.820	0.943	2.060	0.075	0.092
		Sample 22	102502	259	267.55	16.09	0.94	3.75	37.82	7	0.436	0.676	0.062	0.178	0.313	0.202	0.275	7182	0.202	0.062	0.747	0.925	2.059	0.092	0.114
Fluoride application	Baseline	Sample 23	201701	247	255.08	22.22	0.96	3.84	32.92	8	0.571	0.697	0.090	0.160	0.317	0.107	0.205	6364	0.107	0.045	0.773	0.924	2.059	0.099	0.116
		Sample 24	200101	192	209.50	10.06	0.90	3.09	25.31	4	0.750	0.589	0.052	0.143	0.235	0.252	0.357	12589	0.252	0.099	0.890	0.962	2.060	0.060	0.046
		Sample 25	202801	164	176.83	12.98	0.92	3.12	22.11	5	0.608	0.611	0.079	0.134	0.234	0.153	0.283	5634	0.153	0.077	0.841	0.966	2.059	0.047	0.043
		Sample 26	201501	256	268.16	15.44	0.94	3.61	32.98	6	0.552	0.651	0.060	0.138	0.277	0.194	0.277	15009	0.194	0.065	0.769	0.936	2.057	0.086	0.122
		Sample 27	201401	202	213.50	10.97	0.91	3.03	27.09	4	0.511	0.571	0.054	0.142	0.197	0.187	0.357	8766	0.187	0.091	0.890	0.966	2.060	0.056	0.051
		Sample 28	202601	186	205.13	15.05	0.93	3.35	23.60	6	0.678	0.641	0.081	0.137	0.258	0.180	0.267	11240	0.180	0.066	0.855	0.955	2.059	0.060	0.060
		Sample 29	200201	204	216.21	22.12	0.95	3.65	26.30	8	0.637	0.686	0.108	0.124	0.283	0.110	0.204	6728	0.110	0.045	0.676	0.944	2.057	0.063	0.075
		Sample 30	201901	162	165.14	7.36	0.86	2.91	21.79	3	0.738	0.573	0.045	0.146	0.255	0.325	0.422	11987	0.325	0.136	0.856	0.966	2.060	0.052	0.047
		Sample 31	200901	193	205.21	18.11	0.94	3.49	27.09	6	0.626	0.662	0.094	0.151	0.275	0.110	0.213	3713	0.110	0.055	0.783	0.950	2.059	0.062	0.064
		Sample 32	200701	176	187.40	11.02	0.91	3.21	24.78	4	0.662	0.621	0.063	0.149	0.271	0.234	0.357	7047	0.234	0.091	0.843	0.958	2.059	0.055	0.045
		Sample 33	201201	161	180.12	3.44	0.71	2.30	21.77	1	0.869	0.452	0.021	0.144	0.200	0.528	0.591	18680	0.528	0.291	0.888	0.977	2.059	0.036	0.066
	After 6 Months	Sample 34	200702	208	224.43	6.71	0.85	2.77	27.46	3	0.706	0.520	0.032	0.142	0.194	0.342	0.446	18279	0.342	0.149	0.887	0.970	2.061	0.049	0.058
		Sample 35	200902	191	211.00	5.19	0.81	2.49	24.30	2	0.695	0.474	0.027	0.144	0.155	0.405	0.516	25489	0.405	0.193	0.943	0.977	2.060	0.040	0.024
		Sample 36	201502	315	325.22	16.78	0.94	3.78	45.28	6	0.310	0.656	0.053	0.179	0.295	0.154	0.280	7317	0.154	0.060	0.587	0.916	2.061	0.105	0.298
		Sample 37	201702	231	238.92	16.98	0.94	3.52	32.01	7	0.449	0.646	0.074	0.154	0.248	0.169	0.246	7359	0.169	0.059	0.816	0.947	2.059	0.072	0.064
		Sample 38	201402	263	274.50	11.71	0.91	3.41	35.22	6	0.596	0.613	0.045	0.146	0.245	0.250	0.321	15416	0.250	0.085	0.828	0.946	2.059	0.079	0.070
		Sample 39	201202	279	309.00	23.41	0.96	3.86	39.27	8	0.477	0.686	0.084	0.143	0.298	0.121	0.202	5777	0.121	0.043	0.726	0.925	2.060	0.075	0.135
		Sample 40	200202	183	210.35	17.42	0.94	3.40	23.19	7	0.577	0.653	0.095	0.105	0.255	0.134	0.264	8282	0.134	0.057	0.666	0.957	2.056	0.041	0.075
		Sample 41	200101	214	225.67	9.98	0.90	3.15	27.02	5	0.679	0.587	0.047	0.125	0.218	0.269	0.368	20024	0.269	0.100	0.822	0.961	2.056	0.045	0.094
		Sample 42	201902	215	227.16	15.15	0.93	3.52	30.46	6	0.541	0.655	0.070	0.151	0.288	0.156	0.294	5522	0.156	0.066	0.789	0.944	2.059	0.066	0.092
		Sample 43	202802	179	187.00	11.76	0.91	3.23	23.88	5	0.461	0.622	0.066	0.143	0.251	0.207	0.369	8906	0.207	0.085	0.851	0.959	2.058	0.055	0.044
		Sample 44	202602	214	228.06	10.97	0.91	3.20	30.25	4	0.643	0.596	0.051	0.164	0.248	0.197	0.341	7026	0.197	0.091	0.872	0.955	2.060	0.076	0.056

Table S6. The treatment effects on oral disease pathogenic bacteria

	Tooth surface disinfection			Fluoride application		
	Baseline	After 6 Months	Difference	Baseline	After 6 Months	Difference
Cariogenic bacteria						
Streptococcus mutans	0.0336	0.0001	-0.0334	0.009	0.103	0.093
Streptococcus sobrinus	0.0069	0	-0.0069	0	0.003	0.003
Periodontal pathogen						
Porphyromonas gingivalis	0.0262	0.0059	-0.0202	0	0.0105	0.0105
Tannerella forsythia	0.0167	0.0093	-0.0073	0.0166	0.0251	0.0085
Treponema denticola	0.0303	0.0006	-0.0297	0.0136	0.0028	-0.0108
Aggregatibacter actinomycetemcomitans	0.0045	0	-0.0045	0	0.0099	0.0099
Fusobacterium nucleatum group	1.2818	0.7669	-0.5149	0.915	0.9916	0.0766

Table S7. The results of treatment effects on oral disease pathogenic bacteria evaluated by mixed effect modeling

	Streptococcus mutans		*Streptococcus sobrinus*		*Aggregatibacter actinomycetemcomitans*		*Porphyromonas gingivalis*	
	Coefficient	p-value	Coefficient	p-value	Coefficient	p-value	Coefficient	p-value
Intercept	0.072(0.011-0.132)	0.022	1.005(0.996-1.014)	0	0.007(-0.013-0.027)	0.469	0.014(-0.011-0.04)	0.272
Tooth surface disinfection	-0.097(-0.119--0.076)	0	-0.003(-0.007-0.001)	0.165	-0.01(-0.017--0.002)	0.013	-0.003(-0.023-0.017)	0.765
Time	0.026(-0.031-0.083)	0.363	-0.002(-0.01-0.006)	0.622	0.003(-0.016-0.021)	0.774	-0.005(-0.026-0.017)	0.659
AICC	92.909		-253.08		-184.78		-127.529	
BIC	101.51		-244.479		-176.179		-118.928	
Probability distribution	Ganmma		Normal		Ganmma		Ganmma	
Link function	log		identical		log		log	

	Fusobacteriumnucleatum group		*Treponema denticola*		*Tannerella forsythia*	
	Coefficient	p-value	Coefficient	p-value	Coefficient	p-value
Intercept	0.059(-0.388-0.507)	0.791	1.023(1.002-1.044)	0	1.023(1.002-1.044)	0
Tooth surface disinfection	0.06(-0.468-0.588)	0.818	-0.002(-0.006-0.002)	0.284	-0.002(-0.006-0.002)	0.574
Time	-0.214(-0.743-0.316)	0.42	-0.02(-0.041-0.001)	0.058	-0.02(-0.041-0.001)	0.942
AICC	130.727		-197.589		-206.19	
BIC	139.328		1.019		-197.589	
Probability distribution	Ganmma		Normal		Normal	
Link function	log		identical		identical	

Table S8. Detailed correspondence table of allocation numbers and bacterial species in correlation heatmap

Distributed numbers	Corresponding bacterial species	Distributed numbers	Corresponding bacterial species	Distributed numbers	Corresponding bacterial species
1	Abiotrophia defectiva	94	Fusobacterium periodonticum group	187	Prevotella buccae
2	Abiotrophia_uc	95	Fusobacterium_uc	188	Prevotella denticola
3	ACIQ_s	96	Gemella asaccharolytica	189	Prevotella enoeca
4	Actinobaculum timonae	97	Gemella haemolysans group	190	Prevotella fusca
5	Actinomyces bowdenii	98	Gemella morbillorum	191	Prevotella histicola
6	Actinomyces dentalis	99	Gemella palaticanis	192	Prevotella intermedia
7	Actinomyces georgiae	100	Gemella_uc	193	Prevotella jejuni
8	Actinomyces gerencseriae	101	Granulicatella adiacens group	194	Prevotella loescheii
9	Actinomyces graevenitzii	102	Granulicatella elegans	195	Prevotella maculosa
10	Actinomyces hongkongensis group	103	Granulicatella_uc	196	Prevotella marshii
11	Actinomyces israelii	104	Haemophilus haemolyticus	197	Prevotella melaninogenica
12	Actinomyces massiliensis	105	Haemophilus influenzae group	198	Prevotella micans
13	Actinomyces meyeri	106	Haemophilus parahaemolyticus group	199	Prevotella multiformis
14	Actinomyces naeslundii	107	Haemophilus parainfluenzae group	200	Prevotella multisaccharivorax
15	Actinomyces odontolyticus	108	Haemophilus pittmaniae	201	Prevotella nanceiensis group
16	Actinomyces oris	109	Haemophilus quentini	202	Prevotella nigrescens
17	Actinomyces timonensis	110	Haemophilus sputorum	203	Prevotella oralis
18	Actinomyces turicensis	111	Haemophilus_uc	204	Prevotella oris
19	Actinomyces viscosus group	112	Johnsonella ignava	205	Prevotella oulorum
20	Actinomyces_uc	113	Kingella denitrificans	206	Prevotella pallens
21	Aggregatibacter actinomycetemcomitans	114	Kingella oralis	207	Prevotella pleuritidis
22	Aggregatibacter aphrophilus	115	Kingella_uc	208	Prevotella saccharolytica
23	Aggregatibacter segnis	116	Kocuria kristinae	209	Prevotella salivae
24	Aggregatibacter_uc	117	Kocuria palustris group	210	Prevotella scopos
25	Alloprevotella rava	118	KQ969171_s	211	Prevotella shahii
26	Alloprevotella tannerae	119	KV831974_s group	212	Prevotella timonensis
27	Alloprevotella_uc	120	Lachnoanaerobaculum saburreum group	213	Prevotella veroralis
28	Anaerococcus prevotii group	121	Lachnoanaerobaculum umeaense	214	Prevotella_uc
29	Anaeroglobus geminatus	122	Lachnoanaerobaculum_uc	215	Pseudopropionibacterium propionicum
30	Arthrobacter globiformis group	123	Lactobacillus fermentum	216	Pseudoramibacter alactolyticus
31	Atopobium parvulum	124	Lactobacillus gasseri group	217	Pyramidobacter piscolens
32	Atopobium rimae group	125	Lactobacillus helveticus group	218	Rahnella aquatilis group
33	Atopobium_uc	126	Lactobacillus kimbladii	219	Rothia aeria
34	Bacteroides finegoldii	127	Lactobacillus reuteri group	220	Rothia amarae
35	Bergeyella_uc	128	Lactobacillus salivarius	221	Rothia dentocariosa
36	Bifidobacterium bifidum	129	Lautropia mirabilis	222	Rothia mucilaginosa group
37	Bifidobacterium dentium	130	Leptotrichia buccalis group	223	Rothia_uc
38	Bilophila wadsworthia	131	Leptotrichia goodfellowii	224	Ruthenibacterium lactatiformans
39	Bulleidia extructa	132	Leptotrichia hofstadii	225	Saccharimonas_uc
40	Bulleidia_uc	133	Leptotrichia hongkongensis	226	Scardovia wiggsiae
41	CAGY_s	134	Leptotrichia shahii	227	Schwartzia_uc
42	Campylobacter concisus group	135	Leptotrichia trevisanii	228	Selenomonas artemidis
43	Campylobacter curvus	136	Leptotrichia wadei	229	Selenomonas dianae group
44	Campylobacter gracilis	137	Leptotrichia_uc	230	Selenomonas flueggei
45	Campylobacter showae group	138	Massilia aerilata group	231	Selenomonas infelix
46	Campylobacter_uc	139	Massilia arvi group	232	Selenomonas noxia
47	Capnocytophaga gingivalis	140	Megasphaera micronuciformis	233	Selenomonas sputigena
48	Capnocytophaga granulosa	141	Megasphaera_uc	234	Selenomonas_uc
49	Capnocytophaga haemolytica	142	Mogibacterium pumilum group	235	Shuttleworthia satelles
50	Capnocytophaga leadbetteri	143	Mogibacterium timidum	236	Shuttleworthia_uc
51	Capnocytophaga ochracea	144	Morganella morganii group	237	Simonsiella muelleri
52	Capnocytophaga sputigena	145	Moryella_uc	238	Simonsiella_uc
53	Capnocytophaga_uc	146	Mycoplasma faucium	239	Slackia exigua
54	Cardiobacterium hominis	147	Mycoplasma hominis	240	Solobacterium moorei
55	Cardiobacterium valvarum	148	Mycoplasma orale group	241	Staphylococcus aureus group
56	Catonella morbi	149	Mycoplasma salivarium	242	Stomatobaculum longum
57	Centipeda periodontii group	150	Neisseria bacilliformis	243	Streptobacillus felis
58	Clostridium butyricum	151	Neisseria canis	244	Streptobacillus hongkongensis
59	Clostridium magnum	152	Neisseria cinerea	245	Streptobacillus moniliformis group
60	Corynebacterium diphtheriae	153	Neisseria dentiae	246	Streptobacillus_uc
61	Corynebacterium durum	154	Neisseria elongata group	247	Streptococcus anginosus group
62	Corynebacterium kroppenstedtii group	155	Neisseria flavescens	248	Streptococcus constellatus group
63	Corynebacterium matruchotii	156	Neisseria meningitidis group	249	Streptococcus downei group
64	Corynebacterium pseudotuberculosis group	157	Neisseria oralis	250	Streptococcus gordonii group
65	Corynebacterium tuberculostearicum	158	Neisseria perflava	251	Streptococcus mutans
66	Corynebacterium_uc	159	Neisseria sicca group	252	Streptococcus parasanguinis group
67	Cryptobacterium curtum	160	Neisseria subflava	253	Streptococcus parasuis group
68	Cutibacterium acnes group	161	Neisseria weaveri	254	Streptococcus peroris group
69	D89882_s	162	Neisseria_uc	255	Streptococcus pneumoniae group
70	Dialister invisus	163	Oceanivirga salmonicida	256	Streptococcus salivarius group
71	Dialister micraerophilus	164	Oceanivirga_uc	257	Streptococcus sanguinis group
72	Dialister pneumosintes	165	Olsenella profusa	258	Streptococcus sinensis group
73	Eggerthia catenaformis	166	Olsenella uli	259	Streptococcus sobrinus
74	Eikenella corrodens	167	Oribacterium asaccharolyticum	260	Streptococcus_uc
75	Eikenella_uc	168	Oribacterium parvum	261	Tannerella forsythia
76	Enterobacteriaceae group	169	Oribacterium sinus	262	TM7_s
77	Enterococcus_uc	170	Oribacterium_uc	263	Treponema amylovorum
78	Escherichia coli group	171	Ottowia_uc	264	Treponema denticola
79	EU669566_s	172	Parascardovia denticolens	265	Treponema lecithinolyticum
80	EU681966_s	173	Parvimonas micra	266	Treponema maltophilum
81	Eubacterium brachy	174	Parvimonas_uc	267	Treponema medium group
82	Eubacterium infirmum	175	Peptoanaerobacter stomatis	268	Treponema socranskii group
83	Eubacterium nodatum	176	Peptostreptococcus massiliae group	269	Treponema_uc
84	Eubacterium plexicaudatum	177	Peptostreptococcus stomatis group	270	Vagococcus lutrae
85	Eubacterium saphenum	178	Phocaeicola abscessus	271	Vagococcus_uc
86	Eubacterium sulci	179	Porphyromonas catoniae	272	Veillonella atypica
87	Eubacterium yurii group	180	Porphyromonas endodontalis	273	Veillonella caviae
88	Filifactor alocis	181	Porphyromonas gingivalis	274	Veillonella dispar
89	Fretibacterium fastidiosum	182	Porphyromonas pasteri	275	Veillonella parvula group
90	Fusobacterium gonidiaformans group	183	Porphyromonas uenonis group	276	Veillonella rogosae
91	Fusobacterium massiliense	184	Porphyromonas_uc	277	Veillonella_uc
92	Fusobacterium necrophorum group	185	Prevotella aurantiaca	278	Weissella confusa group
93	Fusobacterium nucleatum group	186	Prevotella baroniae		
